# Post-epidemic health system recovery: A comparative case study analysis of routine immunization programs in the Republics of Haiti and Liberia

**DOI:** 10.1371/journal.pone.0292793

**Published:** 2023-10-17

**Authors:** Sanjana J. Ravi, Christina M. Potter, Ligia Paina, Maria W. Merritt

**Affiliations:** 1 The Johns Hopkins Center for Health Security, Johns Hopkins Bloomberg School of Public Health, Baltimore, Maryland, United States of America; 2 Department of International Health, Johns Hopkins Bloomberg School of Public Health, Baltimore, Maryland, United States of America; 3 Johns Hopkins Berman Institute of Bioethics, Baltimore, Maryland, United States of America; University of Ghana, GHANA

## Abstract

Large-scale epidemics in resource-constrained settings disrupt delivery of core health services, such as routine immunization. Rebuilding and strengthening routine immunization programs following epidemics is an essential step toward improving vaccine equity and averting future outbreaks. We performed a comparative case study analysis of routine immunization program recovery in Liberia and Haiti following the 2014–16 West Africa Ebola epidemic and 2010s cholera epidemic, respectively. First, we triangulated data between the peer-reviewed and grey literature; in-depth key informant interviews with subject matter experts; and quantitative metrics of population health and health system functioning. We used these data to construct thick descriptive narratives for each case. Finally, we performed a cross-case comparison by applying a thematic matrix based on the Essential Public Health Services framework to each case narrative. In Liberia, post-Ebola routine immunization coverage surpassed pre-epidemic levels, a feat attributable to investments in surveillance, comprehensive risk communication, robust political support for and leadership around immunization, and strong public-sector recovery planning. Recovery efforts in Haiti were fragmented across a broad range of non-governmental agencies. Limitations in funding, workforce development, and community engagement further impeded vaccine uptake. Consequently, Haiti reported significant disparities in subnational immunization coverage following the epidemic. This study suggests that embedding in-country expertise within outbreak response structures, respecting governmental autonomy, aligning post-epidemic recovery plans and policies, and integrating outbreak response assets into robust systems of primary care contribute to higher, more equitable levels of routine immunization coverage in resource-constrained settings recovering from epidemics.

## Introduction

Acute infectious disease outbreaks are major causes of morbidity and mortality worldwide. Such crises place enormous strains on health systems to both scale up core public health activities to mitigate the threat at hand and absorb escalating demand for emergency medical services. Concurrently, outbreaks also derail routine health service delivery by diverting needed human, financial, and medical resources away from essential health programs. Such strains are especially consequential in resource-constrained settings with large existing burdens of endemic disease, where barriers to accessing basic health services are consistently high, and where even routine care-seeking could result in catastrophic health spending for individual patients and families.

Though the disruptive impacts of major outbreaks on health system performance are well-characterized, the mechanics of *post-*epidemic health system recovery remain somewhat obscure, especially in low- and middle-income countries (LMICs) [1–3]. Emergency planning efforts often prioritize acute response activities (e.g., managing patient surges, reducing disease incidence and mortality) over longer-term health system considerations and population health needs, such as resuming delivery of routine immunization, nutrition, mental health, and reproductive health services [4]. A recent analysis of 154 COVID-19 response plans from 106 countries, for instance, reveals that only 47% included provisions for maintaining essential health services, while only 7% addressed monitoring and evaluation of such services [5]. Recouping losses of health service coverage, rebuilding trust among target populations, and rectifying distortions of national health priorities due to external donor influence are further examples of post-epidemic health system recovery challenges [6]. Underpinning many of these challenges is an imperative for decision-makers to integrate vertically channeled resources for outbreak response into resilient systems for providing routine care.

Routine immunization considerations are particularly important during post-epidemic recovery. Immunization is among the most effective public health interventions, and high levels of coverage are both key to averting outbreaks of vaccine-preventable diseases and an important proxy of strong health system functioning [7,8]. Two recent crises, the 2014–16 West Africa Ebola epidemic in Liberia and the 2010s cholera epidemic in Haiti, serve as instructive–and contrasting–cases of routine immunization program recovery after destabilizing outbreaks. Though national coverage of the first dose of measles-containing vaccine (MCV1) plummeted to 58% in Liberia in 2014 (the first year of its Ebola epidemic), this estimate surged to 87% in 2017, the year after the epidemic was declared over. Similarly, the percentage of Liberian districts reporting over 80% MCV1 coverage increased from 7% in 2014 to 80% in 2018. By contrast, unfortunately, national MCV1 coverage in Haiti stagnated between 64–69% during its years-long cholera epidemic (2010–2019), while the proportion of districts with MCV1 coverage above 80% fell to as low as 21.43% during this period [9].

This study examines the factors that contributed to these differing trajectories, particularly those related to public health practice during and after each country’s epidemic. By comparing the post-epidemic measures implemented in each setting, we identify facilitators and barriers of efforts to rebuild and strengthen routine immunization programs.

## Methods

We performed a case study analysis to explore efforts to integrate outbreak response assets into routine immunization programs in LMICs and improve post-epidemic routine immunization coverage. We utilized a comparative “most-similar” design, wherein we examined two distinct cases that are similar across relevant background conditions but differ across select independent variables and the outcome of interest [10].

### Case selection

The two cases chosen were the 2014–16 West Africa Ebola epidemic in Liberia and the 2010s cholera epidemic in Haiti. These cases were selected by reviewing WHO’s Disease Outbreak News archive of notifiable events reported by country and year (reviewed in October 2019) [11]. To ensure availability of relevant, recently published literature and data, we sought high-profile, rapid onset, naturally occurring infectious disease epidemics that took place in LMICs before 2010, resulted in considerable morbidity and mortality, overwhelmed the health system capacities of affected countries, demonstrated potential for transnational spread, and required significant international intervention and coordination to mitigate.

We determined that the 2014–16 Ebola epidemic in Liberia and the 2010s cholera epidemic in Haiti were the most comparable cases. Both are small, low-income countries with similar governance structures: they are representative, democratic, presidential republics with bicameral legislatures [12,13]. Their national health systems share a similar structure, being comprised of primary-level clinics, service delivery points, and community-based health centers offering basic preventive and curative services; secondary-level hospitals providing emergency, diagnostic, and surgical services; and tertiary-level facilities providing specialized surgical services and advanced care for non-communicable conditions [14,15]. Both countries rely heavily on foreign aid and external donors to subsidize basic health service provision [16,17]. Finally, the Ebola and cholera epidemics were both preceded by major destabilizing events that drastically weakened health system capacities: in Haiti, a catastrophic earthquake, and in Liberia, the First and Second Liberian Civil Wars. Yet, as previously noted, the World Health Organization (WHO) and UNICEF report contrasting trends in post-epidemic routine immunization coverage between the two countries–a feature of these cases enabling theoretical replication [18].

Our chosen unit of analysis in each case was the post-epidemic process of immunization program recovery at the national level, whereby resources channeled vertically toward outbreak response efforts were leveraged with varying degrees of success to increase routine immunization coverage.

### Data collection

Data were gathered from and triangulated among three sources to ensure credibility: 1) peer-reviewed and grey English-language literature; 2) publicly available quantitative databases; and 3) key informant interviews with subject matter experts (see Discussion for further consideration of methodological strengths and limitations).

First, we performed a rapid review of the peer-reviewed, English-language literature in PubMed and Scopus, identifying scholarly analyses of routine immunization challenges in Liberia and Haiti both during and after the Ebola and cholera epidemics, respectively. Rapid reviews–also known as rapid evidence assessments–involve a critical appraisal of what is already known about the topic of interest, with the goal of informing decision-making in a timely, efficient manner [19,20]. Such assessments typically seek to examine the impact of a given variable or intervention, or the antecedents of a given outcome, which suited the purpose of this case study analysis [21]. The results were narrowed to papers published between the ten years prior to each country’s epidemic and the present day. Additionally, we purposively scanned the grey literature to identify technical reports, whitepapers, and other relevant documents relating to post-epidemic routine immunization funding, programs, and activities in both countries. We also applied forward- and backward-snowballing methods (i.e., electronic citation tracking and parsing the references of initially identified sources, respectively) to identify additional relevant documents. Further details of the search strategy (i.e., search terms and document libraries) are available in S1 Appendix.

Next, we obtained quantitative data describing vaccination coverage, immunization and other health system capacities, health spending, and humanitarian and foreign aid disbursements in both countries. Given limited availability of quantitative data for each country, we focused our search on the five years preceding and following the year in which each country’s epidemic began. A list of specific databases consulted and measures collected is also available in S1 Appendix.

The third arm of data collection involved semi-structured key informant interviews who currently or formerly worked in the health or humanitarian response sectors in either Liberia or Haiti during or after each country’s respective epidemic. We sought key informants with expertise in routine immunization, infectious disease epidemiology, outbreak response, health systems-strengthening, community health and primary care, policy, or governance. Drawing from the literature review, *a priori* knowledge, and the Essential Public Health Services framework (see “Data Analysis” below), we developed a preliminary interview protocol to help guide discussions with key informants (S2 Appendix). Next, we identified potential interviewees from our rapid review, existing professional networks, and via snowballing (i.e., soliciting recommendations from confirmed interviewees). We sent interview invitations to 82 individuals via email; ultimately, a total of 21 individuals agreed to participate, including 10 informants with expertise on Haiti and 11 experts on Liberia. A descriptive, de-identified roster of confirmed key informants is provided in S3 Appendix.

The interviews took place between June 7-August 5, 2021, ranged between 45 minutes to an hour each, and were conducted in English. Interviews proceeded on a not-for-attribution basis to encourage frank conversation, and participants provided both written consent via email and oral consent prior to recording. One member of the project team led all the interviews, which were held and recorded via video conference or phone. A second team member took detailed notes on each call, and all recordings and notes were archived on a password-protected server accessible only to the project team. We ceased soliciting additional informants once no significant or novel findings emerged from subsequent interviews.

### Data analysis

**Fig 1:**
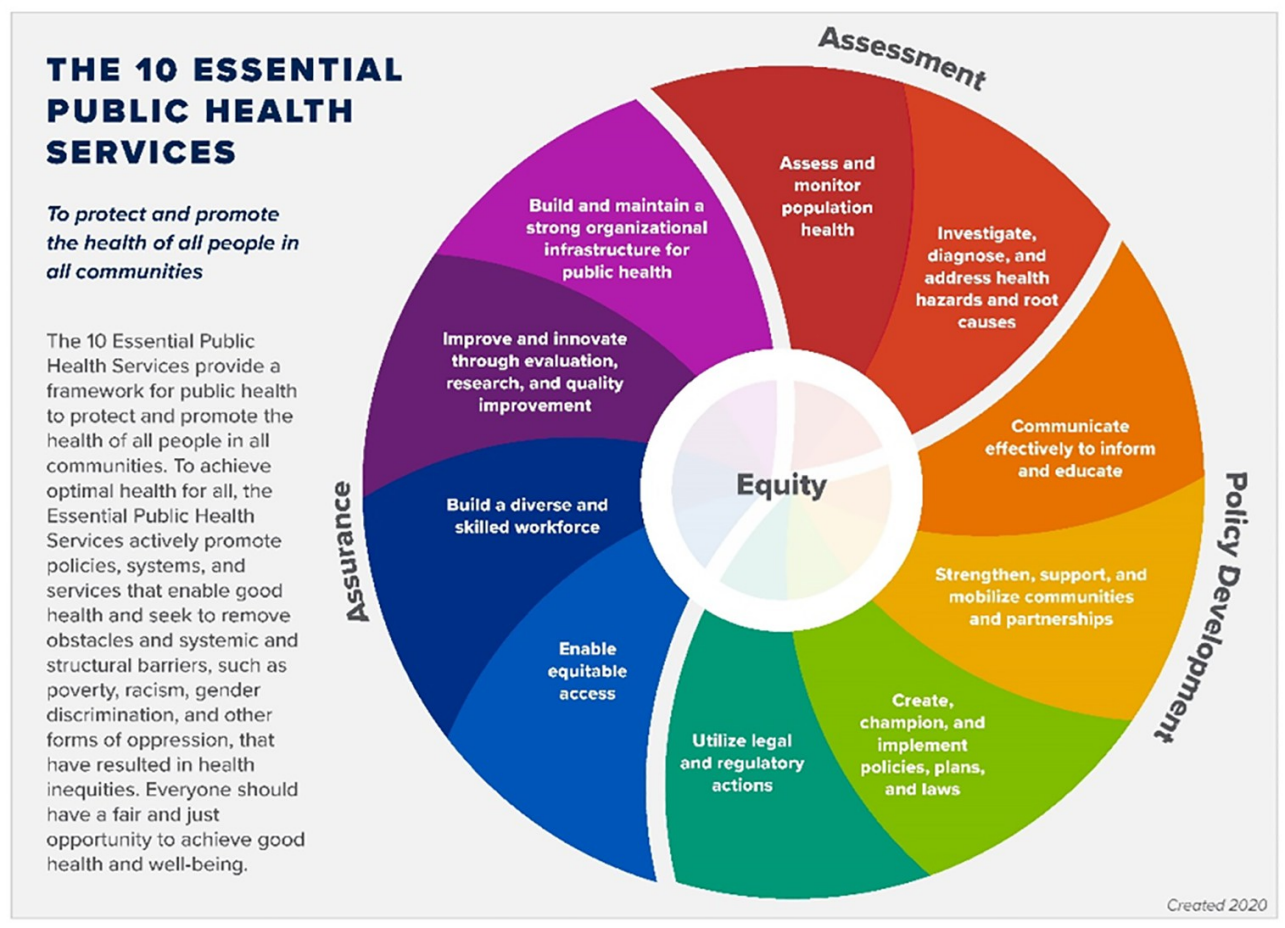
The 10 Essential Public Health Services (courtesy of the U.S. Centers for Disease Control & Prevention and the de Beaumont Foundation).

First, we informally surveyed the literature to identify potential analytical frameworks with which to perform cross-case comparisons. The revised Essential Public Health Services framework (“the Framework”) emerged as a possible cross-case comparative tool (see Fig 1) [22]. Fitter et al. have applied this framework to examine post-earthquake and -cholera health system recovery in Haiti, while others have described it as a roadmap for post-disaster community revitalization [23–25]. Additionally, the Public Health National Center for Innovations and the de Beaumont Foundation recently led an effort to revise the Framework to more explicitly center health equity considerations [26]. For these reasons, we regarded the revised Framework as a suitable tool for comparing post-epidemic immunization program recovery trajectories in Liberia and Haiti.

We coded the literature and interview notes in NVivo 12, using the components of the Framework as our coding scheme. Additionally, during the coding process, we iteratively identified new themes that were not articulated in the original Framework: the roles of colonialism in shaping health systems, country ownership of health programs, and the role of health service integration in post-epidemic recovery. [27]. Upon completion of coding, we used the Framework as a cross-case analytical matrix to order data abstracted from each case and examine similarities and differences between cases across the thematic codes (i.e., “stacking comparable cases”) [28]. By critically reviewing the completed matrix for similarities and differences between the two cases, we identified factors that facilitated or impeded equitable vaccination coverage in post-epidemic Liberia and Haiti. As part of the cross-case analysis, we present relevant mean values for our quantitative measures (i.e., national immunization coverage estimates and health expenditures), in addition to incorporating other quantitative measures throughout each case narrative to illustrate trends in pre- and post-epidemic health system functioning in each country. We also used Stata 17 to perform one-way repeated measures analyses of variance (ANOVA) to assess the potential significance of changes in these measures during the five years preceding and following the year of epidemic onset in each country [29].

Finally, to ensure the transferability of our findings, we constructed thick descriptive narratives for each case, incorporating findings from the literature, interviews, and quantitative databases [30]. Quotes from key informants were verified by reviewing interview recordings. As it did not qualify as human subjects research, this investigation was deemed exempt from full review by the Institutional Review Board at the Johns Hopkins Bloomberg School of Public Health (FWA #00000287).

### Reflexivity statement

At the time of writing, the study team consisted of a recent doctoral graduate (SJR), a junior scholar (CMP), a health systems researcher (LP), and a public health ethicist (MWM), all of whom are employed by a large research university in the United States. SJR adopted a pragmatic lens to conceptualize the study and while leading the data collection and analysis activities described above. To foster reflexivity, SJR and CMP met routinely over the course of the investigation to review study notes and data, discuss emergent findings, and reflect on researcher positionality. LP and MWM provided critical feedback on the study design, data analysis, and manuscript development; MWM also supervised the study. None of the authors have worked directly in Haiti or Liberia; however, SJR and CMP have actively engaged with researchers, practitioners, and scholarship from both countries.

## Findings

Throughout each country’s case narrative, we refer to key informants by anonymized alphanumeric identifiers (S3 Appendix). Additional quotes from key informants are presented in S4 Appendix, where they have been mapped against the components of the Framework. We also provide narratives of key historical events in Haiti and Liberia in S5 Appendix. These historical summaries are meant to provide relevant contextual details and inform broader understandings of health system functioning, particularly with respect to how colonialism and imperialism have shaped population health and public health practice in each country.

### Haiti

A 7.2 magnitude earthquake struck Haiti on January 12, 2010, displacing millions across the country. The official death toll has been disputed, with some estimates placing the number of deaths at 158,000, while the Haitian government reports as many as 316,000 lives lost [31,32]. The earthquake also destroyed an estimated 250,000 homes and 30,000 businesses, sparking protests over the initially sluggish influx of emergency assistance [33].

A Post-Disaster Needs Assessment (PDNA) performed by the United Nations Development Programme highlighted devastating impacts on the country’s already-tenuous health system. Prior to the earthquake, stark inequities were reported in health service access and utilization, reflecting the impact of historical and political chaos on its health system. Maternal and infant mortality rates in 2010 were reported at 630 deaths and 57 deaths per 100,000, respectively; only 6% of the poorest women in Haiti gave birth in a health facility, compared to 65% of the wealthiest; and 47% of the population lacked access to healthcare altogether [34]. In 2013, a few years after the earthquake, only 43% (n = 332) of Haiti’s 786 primary health facilities were deemed to be accessible, while a mere 4% (n = 30) and 6% (n = 42) reported effective service delivery and satisfactory primary care functions, respectively [35]. Furthermore, with a health workforce density of 0.65 doctors, midwives, and nurses per 1,000 people in 2015, Haiti–a country of over 10 million people–falls well below the WHO-recommended minimum health workforce density (4.45 doctors, midwives, and nurses per 1,000) required to achieve the Sustainable Development Goals [36].

**Fig 2:**
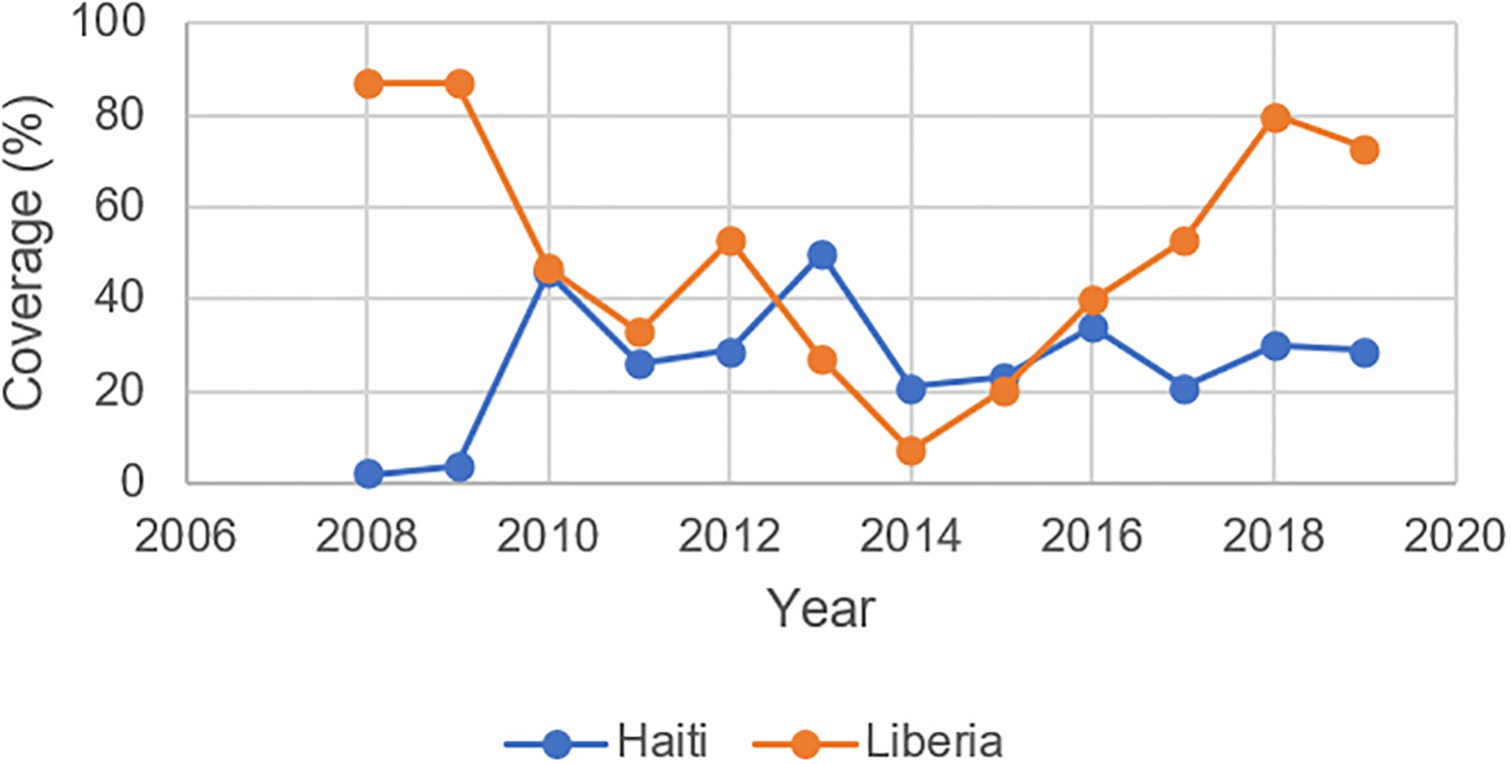
Proportion of districts with at least 80% MCV1 coverage in Liberia and Haiti, 2008–2019.

**Fig 3:**
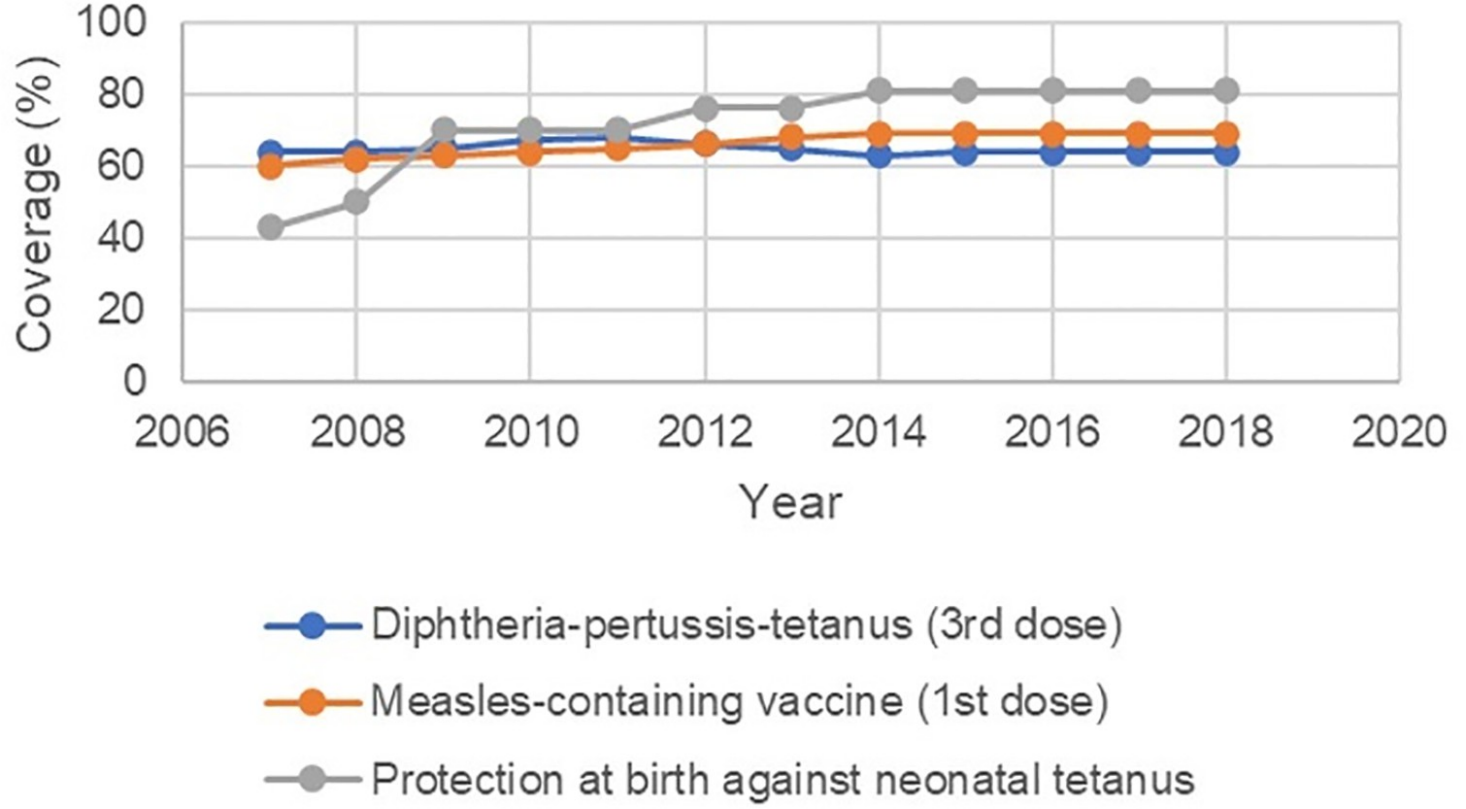
Health service coverage in Haiti, 2007–18.

**Fig 4:**
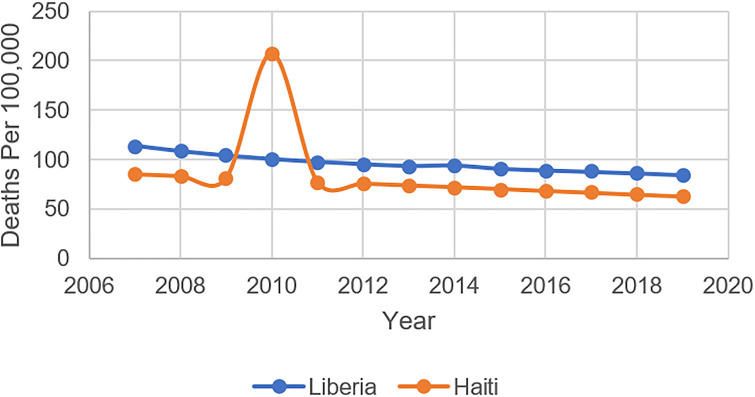
Under-5 mortality in Liberia & Haiti, 2007–19.

Between 2003 and 2009, out-of-pocket costs ranged between 39–48% of the country’s total health expenditures while domestic government health expenditures per capita fluctuated between 6–21%, a consequence of Haiti’s fee-for-service healthcare scheme [37]. In fact, Haiti’s Expanded Programme on Immunization (EPI) estimated that a basic package of health services, including immunization, would cost roughly USD$60 per capita, well above the government’s then-health expenditures of USD$31 per capita [38]. Immunization coverage during this period was also dangerously low: Haiti’s 2005–06 Demographic Health Survey (DHS) reported that a mere 25.7% of children in the wealthiest quintile had received all age appropriate vaccinations, compared to only 9.1% of their poorest counterparts [39]. Figs 2–4 illustrate trends in immunization coverage and under-5 mortality in Haiti during its pre- and post-earthquake periods, drawing from data published in the country’s WHO-UNICEF Joint Reporting Form on Immunization. KI10 described the wide-ranging challenges in administering vaccines after the earthquake: “Vaccinating children was always a struggle…At the micro level, we were able to vaccinate some children in the district, but at the macro level, there were a number of barriers. There are issues with data quality, data collection, planning, and supervision of activities. [The] Ministry of Health has the burden of vaccinating children but there are not sufficient numbers to cover the territory. They were also not well supervised”.

Before 2010, cholera had not been reported in Haiti for over a century [40]. Then, in October of that year, a group of Nepali MINUSTAH peacekeepers arrived in Haiti to support earthquake relief efforts. Epidemiological evidence suggests they had contracted cholera while training in Kathmandu Valley amid an ongoing outbreak, before arriving in Haiti without being screened for the disease [41]. On October 21, Haiti’s National Laboratory of Public Health detected the causative agent, *Vibrio cholerae*, and by October 27, 4,722 cases and 303 deaths were reported [42]. Haiti’s fragile post-earthquake water and sanitation infrastructure catalyzed rapid community transmission. Ultimately, the epidemic–which was finally declared over in 2019 –resulted in roughly 820,000 cases and claimed nearly 10,000 lives [43].

According to KI4, the response to the cholera epidemic mirrored the initial disjointedness of earthquake relief activities, with donors and agencies frequently sidelining Haiti’s ministry of health (Ministère de la Santé Publique et de la Population, MSPP) in favor of commandeering their own response strategies and implementing highly siloed surveillance, healthcare, and vaccination programs. KI4 also observed that the outbreak had immediate impacts on routine health service coverage, recalling, “There was reduced access to essential health services, so paradoxically, it was better to get cholera than any other disease. It mostly had a negative impact on other essential health services. And the staff was redirected as well. All [community health workers, CHWs] working on other stuff were repurposed to cholera outbreaks.”

In 2013, MSPP and its partners launched a USD$2.2 billion strategy to eliminate cholera from Haiti (“National Plan for the Elimination of Cholera in Haiti”) while simultaneously overhauling the country’s public health system; however, by the end of 2014, 190 of the country’s 250 cholera treatment centers had closed and 60% of the remaining facilities lacked adequate infrastructure [44]. The eventual exodus of NGOs from Haiti’s health sector created a vacuum of health systems-strengthening resources and expertise: while as many as 400 organizations initially provided humanitarian aid, only 99 were conducting health activities as of 2011 [45].

In March 2010, MSPP and its partners published an Action Plan for National Recovery and Development. This Action Plan, alongside the country’s PDNA and cholera elimination plan, charted a path for health system recovery and enhancement, focusing primarily on reconstructing health facilities, rebuilding the country’s health workforce, and forging partnerships with the private sector to expand health service delivery. Though the cholera elimination plan briefly alludes to improving “provision of vaccines, cold chains, equipment, and other supplies” and details plans to eventually integrate cholera vaccination into the national childhood immunization schedule, other routine immunization concerns do not figure prominently in any of these recovery strategies [34,46,47].

In contrast to the cholera elimination plan, Haiti’s Expanded Program on Immunization (EPI) program developed a more detailed comprehensive multi-year plan (cMYP) for addressing immunization challenges following the 2010 earthquake. High-level priorities articulated in cMYP include improving immunization program governance; strengthening EPI capacities in rural, peri-urban, and other marginalized regions; and achieving financial sustainability to ensure long-term equitable access, quality, and safety of immunization programs [38]. cMYP also stated that EPI “should be used as a gateway to a minimum package of preventive, promotional and nutritional [services] targeting the dyad of the mother-child,” underscoring the need for integrated approaches to health service delivery [38]. Sufficient funding for these efforts, however, would rely heavily on external partners and donors, including the Pan-American Health Organization (PAHO), UNICEF, Gavi, USAID, the Japan International Cooperation Agency, and Haiti’s tripartite partnership with Cuba and Brazil [38].

MSPP also devised a National Post-Disaster Vaccination Plan with PAHO and UNICEF in 2010, which delineated a phased approach to increasing vaccination coverage and resurrecting routine immunization programs. In accordance with this plan, MSPP launched an integrated campaign in 2010 offering deworming tablets, Vitamin A supplementation, and vaccinations against measles, polio, diphtheria, pertussis, and tetanus. The first phase targeted residents of temporary settlements in the regions most affected by the earthquake, while the second phase expanded to all persons in these areas. By June 2010, over 900,000 people had been vaccinated, including 62% of the total population in the settlements and over 80% of children under five [48]. KI9 highlighted the logistical challenges associated with increasing routine coverage via campaigns, particularly as new vaccines were introduced into Haiti:

“[The campaigns] were a good start to get kids vaccinated in the age range they had to be vaccinated. If you miss that age range in a campaign, they’ll never get it. [But] then there was the push to add all these new vaccines…Then we had to know what rotavirus serotypes and diarrhea surveillance so we could assess impact from vaccination after it was introduced. We had to get cold chain back on board, since many fridges were not functional or destroyed after the earthquake. We had to monitor temperatures in the fridges before we could put vaccine in there. We had to worry about infection spreading in camps early on as well as importation of disease from people coming in.”

Tohme et al. further report that these campaigns achieved lower levels of coverage in the western, central, and Port-au-Prince metropolitan regions of the country, due to children being away from home when vaccinators arrived, or to caregivers being unaware of the campaign [49]. Gavi also reported that a lack of proactive communication and messaging around the importance and value of vaccination–combined with poor patient experiences with healthcare providers–further undermined vaccine demand among the campaign’s target populations [50]. Gavi further notes that forging public-private-civil society partnerships–for example, between the country’s EPI program, the Haitian Society of Pediatrics, the Haitian Red Cross, and the Haitian Platform for Civil Society Organisations to Strengthen Immunisation–could support improved immunization outcomes in the future [50].

Following the campaigns, MSPP embarked upon the third and final phase of the National Post-Disaster Vaccination Plan: jumpstarting the country’s national routine immunization program. KI8 shared that the U.S. Centers for Disease Control and Prevention (CDC), Brazil, Cuba, WHO, and UNICEF partnered with Haiti to place a medical consultant in each department who was tasked with supporting vaccination and surveillance (a department is a subnational administrative division, of which there are ten in Haiti. Haiti’s departments are further divided into 42 arondissements, 145 communes, and 571 communal sections). Though intended to inject expertise into subnational immunization structures, this measure did little to resolve lingering challenges around human resource management and financing: “We lacked staff in place to provide vaccinations and [among] department staff. We didn’t have many strong teams in terms of coordinating vaccination activities,” continued KI8. “These department coordinators were also in charge of other programs, too, like nutrition.” This observation is corroborated by Gavi, which describes insufficient technical coordination between departmental and central-level health authorities, difficulties scheduling regular meetings, and delayed reporting of statistical data during this period [50].

Elaborating further on Haiti’s fiscal woes, KI6 highlighted a fundamental misalignment between Haiti’s national health policy, donor priorities, and political will to conduct budgetary planning as the root cause of the country’s health financing challenges. KI8 also added, “The [immunization] program is owned by the partners. We did not see ownership at the national level, and they were not taking control of the immunization program. This was a big lack in Haiti, and it has to be addressed in order for there to be sustainability. We cannot just leave this problem to the partners.” Both Tohme et al. and Dowell et al. echo this observation, underscoring the need to institutionalize immunization expertise and increase government health spending in Haiti, particularly amid competing donor priorities [51,52]. In some cases, however, MSPP appears to have been deliberately excluded from decision-making, as KI1 shared:

“A lot of immunizations, routine or campaign, are being staffed by internationals, and very often, they do not speak Creole or French…If you are working on the ground–anything underneath the most senior level–you need to speak French or Creole…I’ve sat in meetings that were done in Spanish with no [MSPP] present, and they were taking key decisions within the public health sector for immunization…It’s extremely important that the language and cultural fit [are] there.”

The fragmentation of Haiti’s health governance across a vast constellation of stakeholders–each with varying agendas, resources, and levels of capacity–immediately sparked implementation challenges on the ground, particularly with respect to surveillance. Tohme et al. report that Haiti, with assistance from CDC, began scaling up surveillance systems in 2012, establishing a case-based system to monitor measles and rubella; four sentinel surveillance sites for rotaviral diarrhea; another sentinel surveillance system for meningitis; and an environmental surveillance system for poliovirus [51]. These efforts proved fruitful: by 2015, Haiti moved from being ranked the fourth-worst performing country in the Latin American and Caribbean region to the sixth-best in terms of investigating suspected cases of measles and rubella [51]. However, governance and funding challenges continued to undermine surveillance efforts [51,53].

Haiti also made significant progress in bolstering its public health laboratories. Its Laboratoire National de Santé Publique, established in 2006, initially lacked a strategic plan and focused primarily on HIV diagnostic capacities [53]. With support from the U.S. government, Haiti transformed its nascent laboratory network into a tiered, pyramidal system with expanded confirmatory testing capacities, a national strategic plan, training curricula for laboratory personnel, and forthcoming legal and regulatory frameworks for licensing, accreditation, and quality management [53,54]. However, with the near-entirety of the network’s funding coming from the President’s Emergency Plan for AIDS Relief–and the need to outsource equipment maintenance and repair to external contractors–the sustainability of this laboratory system remains uncertain [53].

In addition to laboratory and surveillance challenges, data deficits hindered routine immunization microplanning (i.e., activities to specify target populations for immunization, identify potential barriers to uptake, and develop workplans for vaccine delivery), vaccine forecasting, and measurement of vaccination coverage. KI3 reported that microplanning is largely supported by UNICEF and takes place at individual health facilities. “I have an Excel sheet and if you click on the name of the facility, you have all the information about microplanning,” they explained. “Microplanning is really well done, but the target population is the issue. Knowledge, skill, and experience goes into great planning, but if you don’t know the number of people you’re serving, your great microplanning doesn’t matter.”

Like many resource-constrained settings, Haiti struggles to estimate health service coverage due to outdated target population estimates [55]. Concurrently, while inaccurate population estimates compromise the denominator of a coverage calculation, poor documentation of vaccinations administered distorts the numerator, thereby producing artificially low coverage estimates. “To speak clearly, we don’t what vaccination coverage is, actually,” admitted KI3. “There is a data issue with big hospitals vaccinating lots of children and not reporting in the database. I know one hospital that vaccinates thousands of children per year that doesn’t report.”

Unreliable coverage estimates, in turn, complicate efforts to forecast vaccine supply and demand, a potentially costly challenge. “It’s also not how many [vaccines] you need, really,” KI1 pointed out. “It’s how many you can use, because if you don’t have a cold system, then what is going to be your trade-off? How many vaccines do you dare to distribute? Because you get an issue vaccinating with ineffective vaccine–with water–or you have to risk destroying vaccine, which is expensive. Even if the vaccine isn’t expensive, the logistics of getting it from the capital to the countryside is expensive.” Gavi further reports that insufficient data-sharing between departmental- and central-level authorities impedes immunization planning, “thereby not encouraging the emergence of strategies to improve immunisation coverage and equity in Haiti” [50].

Finally, workforce challenges–particularly compensation and job satisfaction–were major barriers to improving routine immunization. “Salaries are really low in the public sector, and it’s really a lot of work,” shared KI2, adding that “sometimes, at a health institution, you have very little staff expected to do everything. Any time something new comes in, then it’s added work. So, there’s constantly issues with workload unrelated to compensation and that does affect satisfaction and that’s at all levels from entry-level to management.” The near-constant, kaleidoscopic shifting of Haiti’s donor landscape has further destabilized the country’s health sector job market and fueled health worker discontent. MSPP’s overreliance on donors, for example, has triggered considerable health worker attrition from the public sector since private sector entities generally offer better wages and benefits [56]. Hashimoto et al. also report health worker dissatisfaction with placement in remote geographic areas, high turnover, and chronically delayed payments as additional challenges plaguing Haiti’s public sector health workforce [57].

### Liberia

In December 2013, an index case of Ebola virus disease (EVD) was diagnosed in an eighteen-month-old boy in Guéckédou Prefecture, Guinea [58]. On March 23, 2014, following reports of 49 confirmed cases and 23 deaths in Guinea, WHO declared an outbreak [58]. The very next day, the Liberian Ministries of Information, Culture, Tourism, and Health announced six suspected cases of Ebola in the country, of whom five had already died [59]. Over the next two years, the outbreak rapidly escalated to 10,678 cases and 4,810 deaths in Liberia before authorities officially declared its end on June 1, 2016 [58].

Early in the epidemic, health officials in Liberia and their international partners created an Ebola Task Force to coordinate a swift response. But the Task Force soon proved unwieldy. “When EVD emerged, it took a political dimension in terms of leadership. There wasn’t a technical person providing insight but rather a political structure,” recalled KI14. “There was mixed messaging. There was conflicting information [until] the U.S. was able to intervene by…providing accurate information to the public in regard to EVD prevention and control. Then the government could change its strategy for control and prevention by bringing in and respecting the roles of the local leaders and practitioners.”

Liberian authorities ultimately adopted the Incident Management System (IMS) instead: a standardized protocol developed in the U.S. for coordinating emergency response activities [60]. Liberia chose to coordinate IMS activities through a new, donor-funded emergency operations center–which, after the Ebola epidemic, was used to monitor outbreaks of other vaccine-preventable diseases like meningitis [61,62]. Liberia’s President, Ellen Johnson Sirleaf, communicated frequently with the designated incident manager and established an advisory committee comprised of senior officials and international partners to guide response efforts [60].

KI21 also attributed Liberia’s post-epidemic success in routine immunization to IMS, stating, “The collaboration and coordination was difficult at the beginning of [Ebola]. In [the Ministry of Health and Social Welfare, MoHSW], there was the health coordinating committee, but they were very high level. They weren’t prepared for emergency response; they just [had] meetings. IMS worked, so we wondered if that [could] restructure the health care system, especially with community engagement being so critical.” Similarly, Brault et al. write, that “the Liberian government’s relationship with donors had evolved such that donors no longer drove the agenda, but rather accepted guidance from the government on priority areas and needs that the donors could assist with” [63].

The Ebola epidemic had immediate, destabilizing effects on routine immunization programs. When asked whether immunization activities continued during the early days of the outbreak, KI15 responded with an emphatic “no”: “Nowhere. Schools were closed, campaigns were withheld. Communities were not allowed to converge in large groups and health workers didn’t go into communities. If a mother voluntarily brought in her child for vaccination, then [the] child was vaccinated, but no campaigns took place during EVD.” Clarke et al. corroborate this observation, reporting that all planned outreach, vaccine introductions, campaigns, and supplemental immunization activities were halted. Additionally, MoHSW staff–many of whom were diverted from routine programs to Ebola response activities–were subsequently unable to implement EPI workplans. With fewer than 70% of health facilities open, fear of contracting EVD in healthcare settings also caused public demand for health services to plummet [64].

**Fig 5:**
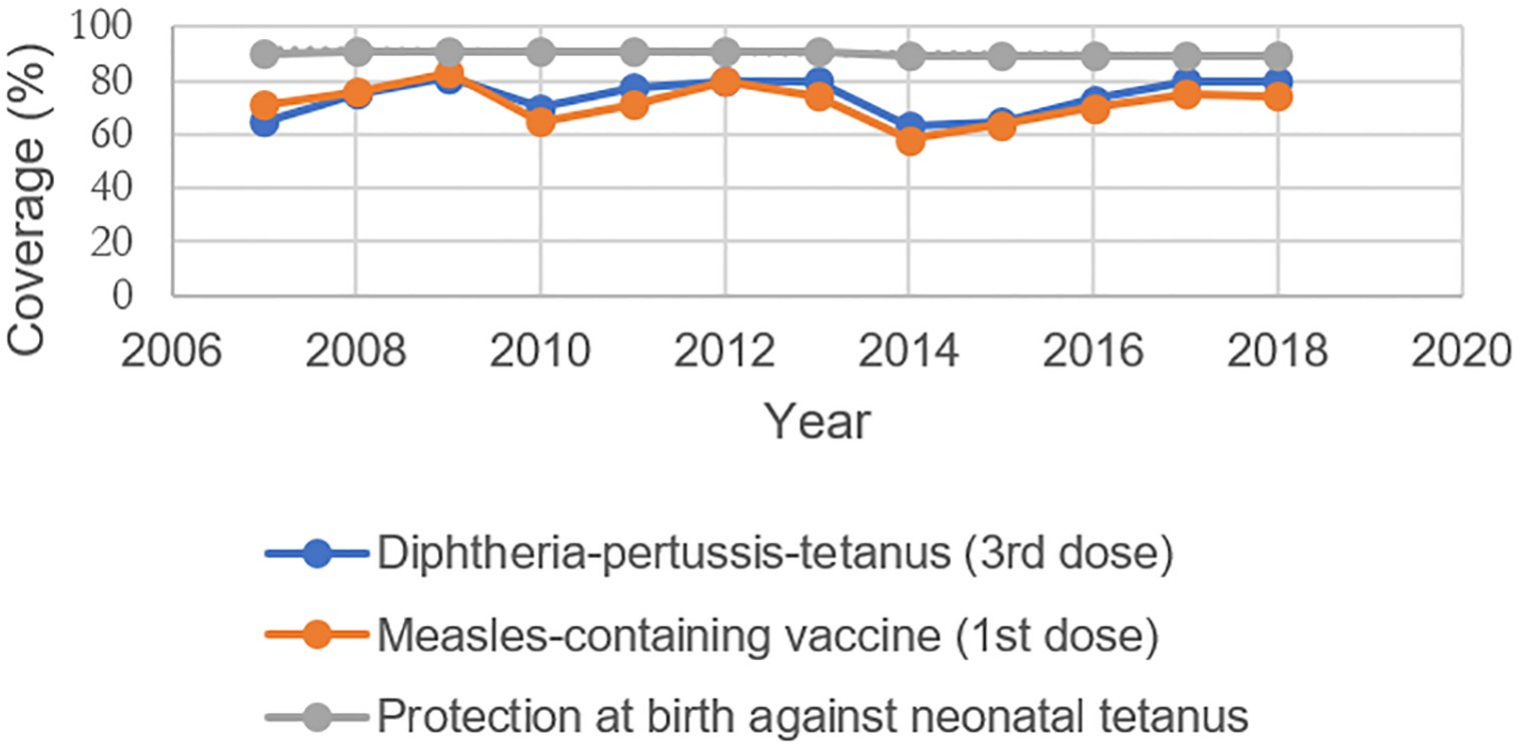
Health service coverage in Liberia, 2007–18.

These setbacks sparked rapid declines in routine immunization coverage across Liberia (see Figs 2 and 5). Vaccination equity also suffered as a result. In 2013, 62.2% of Liberian children aged 24–35 months in the wealthiest quintile and 25.4% in the poorest quintile were fully immunized; in 2016, these estimates fell to 5.2% and 3.7%, respectively. [65] KI17 also highlighted geographic inequities, noting, “The biggest challenge was the issue of equity in immunization…When you dive deeper into the disaggregated data, there were still counties with low coverage.” Wesseh et al. echo this observation, finding that the counties most affected by Ebola (i.e., those reporting more than 70 Ebola deaths) reported 58% fewer fully immunized children during the epidemic; moderately affected counties (10–70 Ebola deaths) and least-affected counties (<10 Ebola deaths) reported reductions of 33% and 39%, respectively [66].

Given these declines in coverage, health authorities prioritized routine immunization activities even during the early days of the epidemic. Liberia’s post-Ebola routine immunization efforts also owe much of their success to proactive planning, effective leadership, and increased investment in health system recovery. As early as December 2014, Liberian health officials participated in a high-level meeting on strengthening health system resilience in Ebola-affected countries, followed by meetings with emergency response stakeholders to achieve consensus on recovery priorities [67]. Next, MoHSW created thematic working groups in alignment with components of WHO’s health system building blocks framework: leadership and governance; service delivery; financing; workforce; medical products, vaccines, and technologies; and information systems [68]. Each working group was comprised of MoHSW senior staff and external partners (including those outside the health sector), and regularly reported to the Cabinet of Liberia. As they liaised with senior MoHSW leaders, the working groups also identified priority areas for Ebola recovery [67].

These planning efforts, in turn, formed a robust policy foundation for post-epidemic health system recovery. Liberia’s Economic Stabilization and Recovery Plan, for example, explicitly connects the country’s economic recovery to health system recovery, reporting that between 2015 and 2017, USD$456 million would be spent on implementing an Investment Plan for Building a Resilient Health System (“the Investment Plan”). Activities included strengthening the health workforce, reengineering health infrastructure, epidemic preparedness and response systems, medical supply and diagnostics management, enhancement of quality delivery systems, information and research management, community engagement, leadership and governance, and financing systems [69].

The Investment Plan and several other policies and strategic plans–the National Health and Social Welfare Policy and Plan 2011–2021 (NHSWP), the National EPI Strategic Plan 2016–2021 (i.e., the comprehensive multi-year plan, cMYP), and the National Action Plan for Health Security (NAPHS)–all feature provisions for strengthening routine immunization services and systems in the wake of Ebola. NHSWP designates clinics as the main delivery points for routine immunization, reaffirms Liberia’s commitment to improving immunization coverage in low-performing jurisdictions via the WHO-endorsed Reaching Every District strategy, and supplementing routine programs with SIAs and National Immunization Days [15]. cMYP, meanwhile, features timelines, objectives, indicators, and cost estimates for increasing immunization coverage, improving cold chain capacities, eliminating stockouts, and strengthening the immunization workforce [70]. NAPHS costs out prioritized capacity-building activities (including roughly USD$4 million for immunization) and echoes cMYP’s commitment to scaling up coverage and human resource capacities [71]. Finally, Liberia’s 1976 Public Health Law–which was revised and approved in 2019 –includes a dedicated chapter on immunization, which specifies leadership roles and responsibilities, articulates guidelines for vaccine management, outlines vaccination requirements, and details financial arrangements for the EPI program [72].

Close alignment and linkages between these foundational documents, policies, and laws facilitated a cohesive approach to resurrecting Liberia’s routine immunization capacities. Yet, despite a strong policy foundation, Liberia’s deteriorating postwar infrastructure continued to hinder immunization. “The lack of infrastructure was terrible there–roads and electricity or lack thereof had the biggest effect on health systems…I’d never seen roads so bad,” recalled KI11. “There used to be electricity and hydro dams and waste treatment in the seventies, but it was all destroyed intentionally during the war. KI19 further underscored the importance of external partner support in overcoming infrastructural challenges, sharing, “With partnership from Gavi, UNICEF, WHO, there were a lot of trainings conducted at both the national and subregional levels, so people knew what to do. Technicians for cold chain were trained and retained and got logistics like motorcycles.” Cold chain challenges had previously encumbered vaccine management and delivery; Gavi, in fact, reported in 2014 that resource-shifting to accommodate the Ebola response caused cold chain equipment to break down, prompting UNICEF to dispatch repair teams [73,74]. Gavi support later enabled Liberia to train a cadre of cold chain officers, construct regional cold stores, and procure cold vans to facilitate vaccine delivery [75]. Between 2016 and 2018, Gavi also subsidized 248 solar direct drive refrigerators to expand the country’s cold chain capacities [75]. These and other Gavi funding streams were instrumental in implementing SIAs, increasing uptake of new and underused vaccines, and supporting urban and non-urban immunization strategies–core components of Liberia’s plan to ensure equitable coverage across all counties [75,76].

Notably, the Ebola epidemic also illuminated critical vulnerabilities in Liberia’s nascent disease surveillance systems. “We realized that we had to make a reform in the human resource structure for surveillance in Liberia,” recalled KI20. “We had to build capacity, look at infrastructure for public health surveillance and diagnostics, and the number of tests that we could conduct in Liberia was far less…Liberia could not perform tests for Lassa, EVD, or yellow fever–a good number of them except for measles.” Experts from MoHSW, WHO, and CDC eventually decided to overhaul Liberia’s Integrated Disease Surveillance and Response (IDSR) platform [77]. WHO originally conceptualized IDSR in 1998 to help African countries strengthen surveillance, laboratory, and response capacities in alignment with the International Health Regulations (2005), and the system had been adopted in Liberia in 2004, albeit without an implementation plan [77,78]. Post-Ebola, however, MoHSW and its partners developed both an implementation plan and a corresponding monitoring strategy [77].

The effort to revitalize IDSR ultimately proved fruitful: improved surveillance, coupled with robust community engagement, facilitated a successful measles immunization campaign among children under ten and improved early detection of both measles and acute flaccid paralysis following Ebola [79,80]. Liberia also adopted the Early Warning and Response Network (EWARN) system, a WHO-prescribed approach for gathering data on acute, rapid onset crises like epidemic-prone disease outbreaks [81]. However, Clarke et al. reported that the presence of parallel surveillance systems running alongside IDSR revealed discrepancies in the data being collected–a challenge also reported in other African countries [64].

Surveillance capacity-building dovetailed with strong commitments to accelerating social mobilization and expanding community outreach during the Ebola epidemic. Community-based surveillance, for example–wherein grassroots informants were enlisted to alert health authorities about suspicious illnesses or deaths–contributed effectively to reducing Ebola transmission [82]. Encouragingly, such protocols were institutionalized in routine public health practice, with health authorities later applying these approaches to measles surveillance and containment [79].

Rebuilding public trust in routine immunization services, after Ebola, however–particularly among the hardest-hit populations–proved to be a daunting challenge [83]. “It was difficult. No one was going up for surveillance or vaccine,” continued KI16. “People were not coming to the health facilities; they were afraid of [healthcare workers, HCWs] infecting the general public. HCWs were also afraid of the general public infecting them.” Liberia’s linguistic diversity further impeded immunization outreach, according to KI11: “People don’t speak the same languages fifteen minutes apart. Entirely different languages, not different dialects…You can’t even sell tomatoes to your neighbors. That makes problems for vaccinators and nurses because they can’t talk to their patients, there’s no common language. How are you supposed to do health education without a common language?”

Confronted with the task of scaling up trust across linguistic barriers, public health authorities coupled mass messaging approaches with grassroots strategies adapted to local contexts. In this vein, KI14 highlighted the role of the country’s postwar peacebuilding infrastructure in supporting health communication, noting that it facilitated dialogue with chiefs in high-risk areas. KI14 also shared that targeted demand generation and awareness-building strategies helped increase vaccine uptake. These included establishing immunization sites in marketplaces, where women with children often worked or shopped; enlisting town criers; broadcasting UNICEF radio programming; and coordinating social media campaigns in support of immunization. KI11 corroborated these examples, sharing, “That was a major thing–building outreach into people’s schedules, linking to [community health workers, CHWs], which were introduced as a formal policy, and pairing with them to plan these outreach events.” On this point, Liberia’s National Community Health Services Policy–originally introduced in 2008, revised in 2011, and renewed in 2016 –established standards for community outreach, health promotion, and referral activities [84].

Importantly, officials also took steps toward strengthening post-epidemic health governance and sustaining political will around immunization. President Sirleaf recognized the gravity of the epidemic early on and prioritized outbreak response efforts, describing Ebola as a threat to the country’s “economic and social fabric” [85]. Writing about Ebola in 2014, she also called for greater investment in Liberia’s health infrastructure, referencing setbacks in routine immunization programming and predicting a resurgence of vaccine-preventable diseases [86]. A strong advocate for community health, President Sirleaf also launched a national health assistance program to serve over 4,000 communities in the remotest parts of the country following the epidemic [87].

The Liberian government further reified its commitments to routine immunization with key structural reforms and financial support. “Because [of] the introduction of yellow fever and pentavalent [vaccines], the Government of Liberia had to commit to co-finance the introduction of these two vaccines, and that has been sustained over the years,” observed KI20. “The government has also demonstrated increased visibility of their support of the immunization program, as demonstrated by budgetary commitments annually to immunization and immunization products.” In this vein, the Investment Plan articulated a strategy to formally ensconce IMS, Ebola-specific community health task forces, and other Ebola coordinating mechanisms within the country’s health sector [88]. The government also strengthened referral mechanisms between Ebola treatment units and the rest of the country’s health system, later developing a plan to decommission these units and transform them into permanent health facilities [88].

Notably, the Liberian government implemented several policy reforms to support post-epidemic recovery. For example, in a departure from the previously established donor-supported financial pooling mechanism for health, the Investment Plan also proposed establishing a government-led equity fund intended to “ensure financial risk protection, cushion against financial risks that limit access to care, and address systemic issues within existing provider payment mechanisms” [88]. MoHSW also provided targeted technical assistance to counties with persistently low levels of coverage–an approach dubbed “Parenting of Poorly Performing Counties” [89].

Though efforts to improve routine immunization coverage following the Ebola epidemic achieved significant progress, several challenges remain. A 2017 audit, for example, showed that IDSR data was not being used to support decision-making at health facilities; many facilities surveyed also reported weak Internet connectivity, electricity outages, and limited access to mobile phones and computers [90]. Additional challenges relate to the management, training, compensation, and retention of Liberia’s health workforce. Here, Liberia reported high attrition among health workers assigned to the southeastern region of the country due to difficult living and working conditions [75]. Additionally, Gavi reported in 2017 that while some 1,110 health workers had been trained in immunization as part of the country’s Ebola recovery efforts, Liberia’s health sector had only 795 working vaccinators, roughly 25% of whom were on government payroll. In fact, as many as 41% of Liberia’s public sector health workers went on strike during the Ebola epidemic to protest their exclusion from the government payroll, prompting the World Bank, African Development Bank, and United Nations Development Programme to help subsidize hazard pay [91].

### Cross-case analysis

#### Analysis of variance

Our one-way repeated measures ANOVA revealed several statistically significant differences between Haiti and Liberia in terms of the proportion of districts reporting 80% or higher coverage of MCV1 (F[10, 9], p = 0.0491), domestic government health expenditure per capita (F[10, 9], p = 0.00), and external health expenditures per capita (F[10, 9], p = 0.0007) during the five years preceding and following their respective epidemics. However, there were no significant differences between Liberia and Haiti in terms of the proportion of districts reporting 80% or greater coverage of DTP3 or dropout rates between DTP3 and the MCV1. In Haiti, the average proportion of districts reporting greater than 80% MCV1 coverage decreased considerably in the five years following the onset of its cholera epidemic. This period also saw a slight decrease in average domestic government health spending and larger increases in average vaccination dropout rates and external health spending. Liberia reported a slightly lower average proportion of districts reporting greater than 80% MCV1 coverage in the five years following its Ebola epidemic, along with a slight decrease in average government health expenditures and a doubling of external health expenditures; notably, average dropout rates during this period also decreased (see S1 Table).

#### Stacked cases

S2 Table–which presents a matrix displaying the findings from each case stacked side-by-side, organized within the Essential Public Health Services framework–facilitates comparison between the post-epidemic recovery experiences of Liberia and Haiti by disaggregating findings across relevant domains of health system functioning (i.e., assessment, policy development, and assurance). Per the Framework, the assessment domain relates to routinely monitoring population health and investigating potential health hazards. Following their respective epidemics, both Haiti and Liberia adopted robust case-based approaches to surveilling vaccine-preventable diseases. In Liberia, however, the IDSR and EWARN systems–coupled with strong case reporting mechanisms, targeted assistance to underperforming counties, and community-based surveillance systems–contributed to better immunization outcomes long after the Ebola epidemic. Though Haiti established a national reference laboratory and developed an accompanying strategic plan, limited availability of equipment and high maintenance costs threaten its fiscal sustainability.

The Framework’s policy development domain encompasses effective communication and health education, community mobilization, policy formulation and implementation, and legal and regulatory measures to promote health. In Liberia, a combination of top-down and grassroots-level communication strategies–coupled with active outreach to last mile communities–played an important role in recouping losses in routine immunization coverage. By contrast, efforts to promote post-cholera routine immunization in Haiti floundered; patient dissatisfaction with immunization service quality at health facilities further diminished public demand for these services. Despite strong relationships with external donors and international organizations, Haiti also struggled to mobilize partnerships with domestic civil society groups well-poised to promote immunization, such as the Haitian Red Cross. Like Haiti, Liberia also sustained strong relationships with international donors; additionally, it excelled at forging partnerships with community champions and civil society organizations, which proved consequential to improving post-Ebola immunization coverage.

The two countries also differed considerably in terms of post-epidemic health system policy formulation. Numerous Liberian policies, strategic plans, and laws demonstrated strong alignment in their vision for a more resilient post-Ebola health system, and several included specific provisions for improving routine immunization. The majority of Haiti’s post-cholera plans and policies, by contrast, did not mention immunization or explicitly articulate strategies for improving coverage beyond the Post-Disaster Vaccination Plan or the EPI program’s cMYP.

The final domain of the Framework, assurance, addresses the importance of ensuring equitable access to needed services, supporting a diverse and skilled health workforce, sustaining a strong organizational infrastructure for public health, and strengthening public health functions through evaluation, research, and continuous quality improvement. Access to immunization services in Haiti was often impeded by violence, user fees, stockouts, long wait times, and lack of referral mechanisms to primary care facilities. Parallel challenges were observed in other realms of public infrastructure in Haiti, where weak transportation and sanitation systems, weak cold chain capacities, and complex land ownership laws hindered efforts to establish permanent mechanisms for vaccine delivery. Though Liberia experienced similar infrastructural challenges, it still managed to strengthen referral mechanisms between Ebola treatment units and primary care facilities, scale up delivery of its Essential Package of Health Services, and establish a National Public Health Institute to inform long-term health systems-strengthening activities.

Despite differing trajectories of post-epidemic immunization coverage, Haiti and Liberia share a critical weakness: health workforce dissatisfaction. Health workers in both countries–including vaccinators and community workers supporting routine immunization programs–were often unhappy with their training, compensation, and geographic placement. In both countries, the epidemic in question diverted health workers from routine service provision, including immunization. Difficult working conditions and more lucrative offers from donor-funded campaigns resulted in health worker attrition from the public sector. Additionally, despite strong microplanning capacities, both countries reported major challenges in data management, estimating immunization coverage, and forecasting vaccine demand. Left unresolved, these shared challenges will likely hinder efforts to achieve and sustain high levels of routine immunization coverage, as well as coordinate new vaccine introductions in the future.

#### Emergent themes

In addition to the domains of the Framework, we identified several other similarities and differences between the two cases relating to the roles of colonialism in shaping health systems, the importance of country ownership and autonomy in matters of health governance, and the role of integration in facilitating post-epidemic recovery (see S4 Appendix for relevant quotes).

Historical injustice is a powerful driver of contemporary inequity. Extractive colonialist practices–military occupation, extortion, distortion of national priorities, and undermining of political authority–have done significant harm to populations and health systems in Haiti and Liberia. Additionally, the centuries-long depletion of social, financial, and political capital initially paralyzed response and long-term recovery efforts in both settings (see S5 Appendix). As political theorist Peer Illner has commented, “[disaster relief] has passed from the domain of state-led, paid reproductive work to the sphere of unwaged reproductive labour. This recent trend has been threefold: exposing communities to disaster by eroding their conditions of life through austerity; abandoning them to survive on their own; then selling off what remains of public relief infrastructures to commercial operators, once the immediate threat has receded” [92]. Illner’s observations mirror the colonial patterns of encroachment and extraction observed in both countries, but especially during the aftermath of the earthquake and cholera in Haiti, wherein nonstate actors commandeered aid flows, outsourced or privatized state capacities, and eschewed government autonomy in deference to donor preferences, all in the name of providing aid and relief.

Both cases also underscored the importance of government autonomy and ownership of health programs as consequential to population health and well-being. As seen in both countries but particularly in Haiti, the implementation of vertical, donor-driven health initiatives in a vacuum of state capacity often weakens public sector agency, credibility, and autonomy; creates an economy of overreliance on external aid to subsidize public goods, including common goods for health; undercuts public trust in a government’s ability to fulfill its social contract; and, in some cases, may actually undermine efforts to develop, operationalize, and institutionalize knowledge, expertise, and capital within the public sector of the recipient country [93]. On this note, Noor adds, “Your help is needed, your ideas are welcome, but the solutions to country problems must be arrived at by countries themselves. Ethical partners know theirs is a supportive role” [94].

Though the literature and interviewees offered critiques of both Liberian and Haitian leadership and donor-driven health initiatives were prevalent in both countries, MoHSW appears to have secured a far greater degree of ownership and autonomy over Ebola response and health system recovery efforts than its MSPP counterparts following the cholera epidemic. By devising a plan to vest control of health financing within its own government and embedding Liberian leaders and experts within donor-funded response and recovery mechanisms, Liberia was able to rapidly scale up national immunization coverage while drastically reducing inequities. The ethos of centering Liberian leadership and technical knowledge shaped nearly all aspects of response and recovery, from revitalizing IDSR and formulating the Investment Plan to implementing IMS and prioritizing community health. Political leaders’ firm commitment to improving immunization and community health capacities–which, in turn, was underpinned by programmatic and financial support–was also instrumental in achieving this. By contrast, the sidelining of MSPP in Haiti and fragmentation of financial and decision-making power across a slew of external actors obstructed cohesive action around immunization. Furthermore, the absence of a unified government response to the cholera epidemic impeded priority-setting, policymaking, and budgeting activities required for long-term immunization program recovery.

From health system ownership and autonomy follows integration: the alignment of health system leaders, priorities, funding streams, policies, workforces, and external stakeholders across a full spectrum of comprehensive health service provision [95]. Liberia’s post-epidemic immunization model–wherein targeted campaigns were not the primary modes of vaccination, but rather supplemented EPHS delivery–resulted in greater coverage compared to Haiti, where standalone vaccination campaigns were implemented largely in isolation from other primary health services. Furthermore, Liberia achieved clear alignment across the various plans, policies, priorities, and budgets guiding immunization program recovery. By contrast, limited planning and budgeting capacities in Haiti, the omission of routine immunization from key strategic plans, and the predominance of external decision-makers and consultants indicate a less-integrated approach to enhancing post-cholera immunization coverage.

## Discussion

This investigation examined the factors contributing to equitable post-epidemic routine immunization coverage in Liberia and Haiti. Lessons learned from past emergencies–infectious disease outbreaks, natural disasters, manmade catastrophes, and others–suggest that health systems should ideally possess the ability to scale up robust horizontal capacities for routine service provision to meet the demands of emergent crises [96]. Yet, the divergent trajectories of post-epidemic routine immunization program recovery in these countries illustrate how such logic often fails in resource-constrained settings, where baseline health system capacities may be weak or missing altogether. We found that achieving strong alignment between immunization policies, integrating routine immunization into robust systems of primary care, and respecting country ownership and autonomy over health system functioning were essential to improving equitable immunization coverage in Liberia. By contrast, the absence of these factors may have contributed to widening coverage disparities in Haiti.

While the specific approaches adopted in these countries may not be universally applicable in every low- or middle-income setting, or after every type of infectious disease outbreak, this study did generate important insights about the facilitators and deterrents of routine immunization program recovery following major epidemics. These insights, in turn, may be transferrable to other resource-constrained settings characterized by weak primary care systems, a heavy reliance on external aid to subsidize both routine and emergency health system activities, and a strong donor presence. Political leaders and domestic health authorities in affected countries should conduct long-term planning to ensure alignment between budgets, plans, and routine immunization programs; treat routine immunization and community health systems as critical national priorities worthy of sustained, long-term investment; strengthen linkages between various health system components; and improve compensation structures and working conditions for public-sector health workforces. Meanwhile, donors and other external stakeholders should strive to embed local expertise and leadership within vertical response and recovery structures, promote country ownership of domestic health programs, and work with in-country political leaders to support long-term budgetary and policy planning around routine immunization, community health, and primary care.

This investigation does have some limitations. First, none of the investigators speak, read, or write French, Haitian Creole, or any non-English language spoken in either Haiti or Liberia with enough proficiency to analyze relevant documents produced in these languages. Similarly, we were unable to interview key informants unless they could converse in English. A second limitation relates to the types of key informants recruited for this study: nearly every interviewee consulted about Haiti was or is currently affiliated with donor agencies that responded to the earthquake and cholera epidemic. However, we were unable to secure many interviews with individuals affiliated with MSPP or Haitian-led organizations. Conversely, most informants interviewed about Liberia were Liberian nationals either currently or formerly affiliated with public-sector institutions that responded to the Ebola epidemic, while few represented donor agencies or external partners. As a result, our findings for both countries do not account for demand-side or end-user perspectives (e.g., from patients) on immunization program recovery, and in the case of Haiti, they do not sufficiently reflect public-sector perspectives (e.g., from MSPP). Furthermore, we identified very few peer-reviewed studies and grey literature documents featuring Haitian authorship; Liberian MoHSW authors, by contrast, were comparatively well-represented in published scholarship on Ebola and routine immunization. Given these omissions, a relatively low response rate among interviewees (21 interviews granted out of 80 invited), and the language barriers, it is likely that valuable perspectives on immunization and health system recovery are missing from both cases–particularly Haitian perspectives. These gaps indicate important areas for future inquiry.

The Essential Public Health Services framework was an intuitive tool in terms of articulating a set of basic organizing principles for health systems and providing a common vernacular for discussing recovery across diverse health system stakeholders. However, it also has some limitations, which became apparent over the course of this investigation. First, the Framework appears to be conceptual in design rather than analytical; as such, it does not indicate how individual essential services should be weighted relative to one another, nor does it suggest how they should be organized, the time required to establish said services, or the order in which stakeholders should pursue them. Furthermore, as Fitter et al. note in their analysis of public health system recovery in Haiti, the Framework does not explicitly account for health governance, political will, leadership, or financing [23]. Perhaps these were envisioned as cross-cutting competencies or considerations rather than essential “services”; if so, a clearer statement of the assumptions underpinning the Framework would be valuable.

Accompanying Framework guidance states, “To achieve equity, the Essential Public Health Services actively promote policies, systems, and overall community conditions that enable optimal health for all and seek to remove systemic and structural barriers that have resulted in health inequities.” The Framework positions equity as the core of the ten essential services but makes no mention of ethics or justice beyond this stated purpose. Nevertheless, by deeming certain services “essential,” the Framework makes implicit normative judgements about their value without explicitly considering structural factors–such as state capacities and colonial legacies–that shape their availability, provision, and utilization. Finally, the Framework does not clearly explicate linkages between individual essential services. Mounier-Jack et al. present similar critiques of WHO’s health system building blocks model–which, like the Framework, does not account for demand-side considerations associated with health service provision or structures of power and decision-making [97]. Thus, practitioners and policymakers might consult the Framework to arrive at a common understanding of health system needs and priorities following a major crisis, but may find it less useful as an operational tool for guiding health system recovery efforts.

To assess the confirmability of this investigation, follow-on case studies might examine post-epidemic immunization programs in other settings to determine whether similar barriers and facilitators of recovery exist. Coupling other methodological frameworks, such as a positive deviance lens, with additional forms of data (e.g., social media content, financial data, mobile data) and modes of data collection (e.g., focus groups, community-based participatory research, surveys) could also yield rich insights into demand-side immunization considerations [98]. Future analyses might also compare perspectives across sectors and stakeholders (e.g., donors, NGOs, public-sector health institutions, patients) to paint a more comprehensive picture of post-epidemic recovery. Additionally, a formal power analysis of post-epidemic health system reforms could elucidate how relationships between donors, policymakers, practitioners, and communities shape population health outcomes following major crises [99].

## Conclusion

As the world continues to navigate the COVID-19 pandemic, our findings may be especially salient for health practitioners working to reverse population health setbacks and ensure the continuity of core public health programs like routine immunization. For example, donors might consider earmarking a portion of emergency response funds toward ensuring the continuity of routine immunization programs and integrating COVID-19 vaccination capacities into primary care systems, while ministerial and subnational health officials should explicitly incorporate these considerations into pandemic response and recovery plans. As Haiti and Liberia’s experiences with large-scale epidemics have shown, decision-makers and donors in LMICs play particularly important roles in course-correcting health systems struggling to meet the demands of public health emergencies, often at the expense of providing core primary health services. How these stakeholders coordinate both intra- and post-crisis recovery efforts will shape future trajectories of population health and health equity.

## Supporting information

S1 Table Pre- & post-epidemic immunization coverage & health spending in Haiti and Liberia. (DOCX)

S2 Table Matrix of stacked cases using the Essential Public Health Services framework. (DOCX)

S1 Appendix Literature and data search strategy. (DOCX)

S2 Appendix Key informant interview protocol. (DOCX)

S3 Appendix Characteristics of key informants interviewed. (DOCX)

S4 Appendix Key informant quotes mapped against components of the Essential Public Health Services framework. (DOCX)

S5 Appendix Historical contexts of Haiti and Liberia. (DOCX)
