# Supplementary material for: Post-epidemic health system recovery: A comparative case study analysis of routine immunization programs in the Republics of Haiti and Liberia
Source: PLoS One. 2023 Oct 17;18(10):e0292793. doi: 10.1371/journal.pone.0292793 (PMC10581452; doi:10.1371/journal.pone.0292793)
Supplement: S1 Table — (DOCX) [file pone.0292793.s001.docx]

| **Table 1. Pre- & Post-Epidemic Immunization Coverage & Health Spending in Haiti and Liberia** | | | | | | | | | | | |
| --- | --- | --- | --- | --- | --- | --- | --- | --- | --- | --- | --- |
| **Haiti** | | | | | | **Liberia** | | | | | |
| **Year** | **Proportion of Districts with ≥ 80% MCV1 Coverage (%)** | **Proportion of Districts with ≥ 80% DTP3 Coverage (%)** | **Dropout Rate Between DTP1 and MCV1 (%)** | **Domestic General Government Health Expenditure per Capita (PPP Int$)** | **External Health Expenditure per Capita (PPP Int$)** | **Year** | **Proportion of Districts with ≥ 80% MCV1 Coverage (%)** | **Proportion of Districts with ≥ 80% DTP3 Coverage (%)** | **Dropout Rate Between DTP1 and MCV1 (%)** | **Domestic General Government Health Expenditure per Capita (PPP Int$)** | **External Health Expenditure per Capita (PPP Int$)** |
| 2005 | 23 | - | -4 | 13 | 25 | 2009 | 73 | 87 | 5 | 35 | 10 |
| 2006 | 21 | 50 | 27 | 6 | 35 | 2010 | 80 | 47 | 15 | 33 | 8 |
| 2007 | 50 | 47 | 30 | 19 | 30 | 2011 | 53 | 53 | 17 | 32 | 16 |
| 2008 | 29 | 29 | - | 20 | 32 | 2012 | 40 | 93 | 22 | 42 | 10 |
| 2009 | 26 | 44 | 18 | 21 | 33 | 2013 | 20 | 87 | 24 | 48 | 5 |
| 2010 | 46 | 68 | 40 | 22 | 56 | 2014 | 7 | 13 | 22 | 46 | 11 |
| 2011 | 4 | 50 | 39 | 11 | 104 | 2015 | 27 | 20 | 17 | 44 | 15 |
| 2012 | 2 | 47 | 30 | 13 | 95 | 2016 | 53 | 80 | 19 | 41 | 19 |
| 2013 | 5 | 57 | 11 | 14 | 59 | 2017 | 33 | 87 | 12 | 35 | 21 |
| 2014 | 9 | 30 | 10 | 14 | 75 | 2018 | 47 | 93 | 8 | 26 | 26 |
| 2015 | - | 32 | 19 | 17 | 78 | 2019 | 87 | 87 | 10 | - | - |
| **Pre-Epidemic** | | | | | | | | | | | |
| **Mean (SD)** | 29.8 (11.7) | 42.5 (9.3) | 17.8 (15.4) | 16 (6.1) | 31 (3.9) | **Mean (SD)** | 53.2 (24.4) | 73.4 (21.6) | 16.6 (7.4) | 38 (6.8) | 10 (4.2) |
| **Post-Epidemic** | | | | | | | | | | | |
| **Mean (SD)** | 5 (1.6) | 43.2 (11.7) | 21.8 (12.5) | 14 (2) | 82 (17.6) | **Mean (SD)** | 49.4 (23.5) | 73.4 (40.2) | 13.2 (4.7) | 36 (8.2) | 20 (4.5) |
