# Supplementary material for: Post-epidemic health system recovery: A comparative case study analysis of routine immunization programs in the Republics of Haiti and Liberia
Source: PLoS One. 2023 Oct 17;18(10):e0292793. doi: 10.1371/journal.pone.0292793 (PMC10581452; doi:10.1371/journal.pone.0292793)
Supplement: S2 Table — (DOCX) [file pone.0292793.s002.docx]

| **Table 2. Matrix of Stacked Cases**  **Using the Essential Public Health Services Framework** | | | |
| --- | --- | --- | --- |
| **Core Function** | **Essential Public**  **Health Service** | **Immunization Program**  **Recovery in Haiti** | **Immunization Program**  **Recovery in Liberia** |
| **Assessment** | Assess and monitor population health status, factors that influence health, and community needs and assets | - Active case-based, sentinel, and environmental surveillance for vaccine-preventable diseases - Limited availability of equipment (e.g., computers) | - Adoption of IDSR & EWARN systems - Strong reporting mechanisms for notifiable conditions - Strong case- and community-based surveillance protocols |
| **Assessment** | Investigate, diagnose, and address health problems and hazards affecting the population | - Creation of a tiered, pyramidal network with a national reference laboratory - Development of a strategic plan and regulatory/legal framework - Funding from PEPFAR - Equipment maintenance outsourced to contractors | - Adoption of IMS structure - Creation of an emergency operations Center - Targeted technical assistance provided to counties with poor immunization coverage |
| **Policy Development** | Communicate effectively to inform and educate people about health, factors that influence it, and how to improve it | - Lack of communication and messaging to raise awareness of the importance of vaccination - Poor patient experiences at healthcare facilities | - Intensified communication efforts via top-down and bottom-up approaches - Active outreach to communities without static clinics - Marketplace-based outreach - Radio programming |
| **Policy Development** | Strengthen, support, and mobilize communities and partnerships to improve health | - Strong relationships with international donors - Lack of formal partnerships between the EPI program and civil society groups (e.g., Haitian Red Cross) | - Strong relationships with international donors - Contracting with civil society organizations to support community engagement - Coordination with tribal chiefs, elders, women groups, and other community champions |
| **Policy Development** | Create, champion, and implement policies, plans, and laws that impact health | - cMYP 2011-2015 - Dedicated National Post-Disaster Vaccination Plan - PDNA and cholera elimination plans do not mention routine immunization | - Political prioritization of immunization - National Health and Social Welfare Policy and Plan - National EPI Strategic Plan - Investment Plan for Rebuilding a Resilient Health System - National Health and Social Welfare Policy & Plan - National Community Health Services Policy - Economic Stabilization and Recovery Plan - National Action Plan for Health Security - National Health and Social Welfare Financing Policy & Plan |
| **Policy Development** | Utilize legal and regulatory actions designed to improve and protect the public’s health | - No national legislation on immunization - Highly centralized organization of public sector health programs | - Public Health Law (2019) - Improved accountability and partner coordination within a decentralized health system structure |
| **Assurance** | Assure an effective system that enables equitable access to the individual services and care needed to be healthy | - Lack of referral mechanisms between cholera treatment units & primary care - Long distance to health facilities; long wait times; inaccessible transportation - Gang violence - Vaccine stockouts - Fee-for-service scheme | - Essential Packages of Health Services - Referral mechanisms strengthened between Ebola treatment units and routine health facilities - Urban Immunization Strategy - Reaching Every District |

| **Assurance** | Build and support a diverse and skilled public health workforce | - Shortage of qualified primary health workers in the public sector - Low, delayed compensation for public sector health workers - Attrition of health workers from the public sector | - Increased immunization workforce training - Expansion of Field Epidemiology Training Program efforts - Low, delayed compensation for public sector health workers - Health worker strikes - Attrition of health workers from the public sector - Need for health worker educational reforms |
| --- | --- | --- | --- |
| **Assurance** | Improve and innovate public health functions through ongoing evaluation, research, and continuous quality improvement | - Strong microplanning capacities - Efforts made to learn from prior disasters in Africa & Asia - Poor census data & unreliable coverage estimates - Weak data management and data-sharing mechanisms | - Strong microplanning capacities - Major improvements in infection prevention & control - Implementation of quality improvement measures at health facilities - Data discrepancies across parallel information systems - Poor census data & unreliable coverage estimates |
| **Assurance** | Build and maintain a strong organizational infrastructure for public health | - Weak public infrastructure (roads, transportation, sanitation systems, energy, buildings, etc.) - Weak cold chain capacities - Few functional health facilities; reliance on temporary structures (e.g., tents) - Land ownership challenges - Parallel, donor-created information systems with no integration - Insufficient staffing at health posts | - Transitioning Ebola treatment units into health facilities - Improved staffing at facilities - Major expansion of cold chain capacities - Established a National Public Health Institute |
