# Supplementary material for: Post-epidemic health system recovery: A comparative case study analysis of routine immunization programs in the Republics of Haiti and Liberia
Source: PLoS One. 2023 Oct 17;18(10):e0292793. doi: 10.1371/journal.pone.0292793 (PMC10581452; doi:10.1371/journal.pone.0292793)
Supplement: S1 Appendix — (DOCX) [file pone.0292793.s003.docx]

**Appendix A: Literature and Data Search Strategy**

**Rapid Review:**

The results of our rapid review were narrowed to papers published between the ten years prior to each country’s epidemic and the present day, and the following terms were used to search both PubMed and Scopus:

- “Liberia” AND “routine immunization” OR “immunization” OR “vaccination”
- “Haiti” AND “routine immunization” OR “immunization” OR “vaccination”

We also purposively scanned the grey literature to identify technical reports, whitepapers, and other relevant documents relating to post-epidemic routine immunization funding, programs, and activities in both countries. These documents and their sources include:

- Joint Appraisal Reports (Gavi, the Vaccine Alliance, “Gavi”)
- Comprehensive Multi-Year Plans for Immunization (Gavi)
- Foreign aid records (U.S. Agency for International Development, “USAID”)
- UN Digital Library (United Nations, UN)
- Multi-Partner Trust Fund Office Gateway (United Nations)
- Development Experience Clearinghouse (USAID)
- Joint External Evaluations (World Health Organization, “WHO”)
- Post-Disaster Needs Assessments (UN Development Programme)
- National Action Plans for Health Security (WHO)
- ReliefWeb search results

We also used forward- and backward-snowballing methods (i.e., electronic citation tracking and parsing the references of initially identified sources, respectively) to identify additional relevant documents.

**Quantitative Data:**

Next, we obtained quantitative data describing vaccination coverage, immunization and other health system capacities, health spending, and humanitarian and foreign aid disbursements in both countries, focusing on the five years preceding and following the year in which each country’s epidemic began. These data and their corresponding sources are presented below.

- WHO-UNICEF Joint Reporting Forms
  - Percentage of districts with microplans to raise immunization coverage Percentage of districts with MCV1 coverage ≥ 80%
  - Percentage of districts with coverage of the third dose of diphtheria-tetanus-pertussis-containing vaccine (DTP3) ≥ 80%
  - Drop-out rate between the first dose of diphtheria-tetanus-pertussis-containing vaccine (DTP1) and MCV1
- WHO Global Health Expenditure Database
  - Domestic general government health expenditure per capita
  - External health expenditure per capita
- ForeignAssistance.gov (USAID)
  - Health sector assistance
  - Humanitarian sector assistance
