# Supplementary material for: Post-epidemic health system recovery: A comparative case study analysis of routine immunization programs in the Republics of Haiti and Liberia
Source: PLoS One. 2023 Oct 17;18(10):e0292793. doi: 10.1371/journal.pone.0292793 (PMC10581452; doi:10.1371/journal.pone.0292793)
Supplement: S2 Appendix — (DOCX) [file pone.0292793.s004.docx]

**Appendix B. Key Informant Interview Protocol**

1. Please describe your involvement in responding to the [Ebola/cholera] epidemic in [Liberia/Haiti].

1. Please describe how your organization coordinated with other NGOs, responding agencies, and government agencies operating in [Liberia/Haiti] during this time.

1. Tell us about what routine immunization activities and programs in [Liberia/Haiti] looked like immediately after the [Civil War/earthquake]. Were there major disruptions?

- 1. What about after the [Ebola/cholera] epidemic took off?
  2. Were there some parts of the country that experienced more disruptions in routine immunization than others?

1. **Assessment:**

- 1. Please describe if or how surveillance for vaccine-preventable diseases changed after the [Ebola/cholera] epidemic took off.
  2. Were there concerns about seeing a spike in measles cases, or cases of other vaccine-preventable diseases? What plans were in place to deal with those, if so?

1. **Policy Development:**

- 1. Can you provide examples of community engagement efforts around routine vaccination during and after the [Ebola/cholera] epidemic? Were there specific risk communication strategies or community partnerships that your team relied on to help promote routine vaccination?
  2. Were there national laws, policies, or other government directives that supported routine immunization during this time?

1. **Assurance:**

- 1. How were resources for routine immunization distributed across [counties, districts, departments, and/or *arondissements*]?
  2. Were there efforts made to strengthen [Liberia/Haiti]’s domestic immunization workforce during this time?
  3. What other steps were taken to strengthen Haiti’s health system during this time?

1. If you could go back in time and change some aspect or aspects of how your organization responded to the crisis in [Liberia/Haiti], what would you change?
