# Supplementary material for: Post-epidemic health system recovery: A comparative case study analysis of routine immunization programs in the Republics of Haiti and Liberia
Source: PLoS One. 2023 Oct 17;18(10):e0292793. doi: 10.1371/journal.pone.0292793 (PMC10581452; doi:10.1371/journal.pone.0292793)
Supplement: S3 Appendix — (DOCX) [file pone.0292793.s005.docx]

**Appendix C: Characteristics of Key Informants Interviewed**

| # | Discipline & Issue Expertise | Professional Domain | | | Geographic Focus | |
| --- | --- | --- | --- | --- | --- | --- |
|  |  | In-Country Government | External Donor or Partner | Civil Society | Liberia | Haiti |
| KI1 | Public health: project management, sustainability, health system governance |  |  | ✓ |  | ✓ |
| KI2 | Public health: immunization, surveillance for vaccine-preventable diseases |  | ✓ |  |  | ✓ |
| KI3 | Public health: maternal & child health, cholera, monitoring & evaluation |  | ✓ |  |  | ✓ |
| KI4 | Humanitarian health: cholera, emergency response, program evaluation |  | ✓ |  |  | ✓ |
| KI5 | Humanitarian health: program coordination, immunization microplanning, outbreak response |  | ✓ | ✓ |  | ✓ |
| KI6 | Health financing: policy, costing of health services, budget management |  | ✓ |  |  | ✓ |
| KI7 | Public health: primary health care, capacity-building, health advocacy |  |  | ✓ |  | ✓ |
| KI8 | Public health: routine immunization |  | ✓ |  |  | ✓ |
| KI9 | Public health: routine immunization, vaccine introduction, surveillance | ✓ |  |  |  | ✓ |
| KI10 | Public health & medicine: primary care, women’s health, health workforce training |  |  | ✓ |  | ✓ |
| KI11 | Public health: epidemiology, monitoring & evaluation |  |  | ✓ | ✓ |  |
| KI12 | Public health: routine immunization, epidemiology, surveillance |  |  | ✓ | ✓ |  |
| KI13 | Public health: demography, incident management, outbreak response | ✓ |  |  | ✓ |  |
| KI14 | Policy: peacebuilding, health governance | ✓ |  | ✓ | ✓ |  |
| KI15 | Public health: routine immunization | ✓ |  | ✓ | ✓ |  |

| # | Discipline & Issue Expertise | Professional Domain | | | Geographic Focus | |
| --- | --- | --- | --- | --- | --- | --- |
|  |  | In-Country Government | External Donor or Partner | Civil Society | Liberia | Haiti |
| KI16 | Public health: routine immunization | ✓ |  | ✓ | ✓ |  |
| KI17 | Public health: routine immunization, epidemiology, child health, medicine |  |  | ✓ | ✓ |  |
| KI18 | Public health: routine immunization, health systems strengthening | ✓ |  | ✓ | ✓ |  |
| KI19 | Public health: community engagement, risk communication | ✓ |  |  | ✓ |  |
| KI20 | Public health: epidemiology, outbreak response, surveillance | ✓ |  |  | ✓ |  |
| KI21 | Public health: incident management, outbreak response, health systems strengthening | ✓ |  |  | ✓ |  |
