# Supplementary material for: Post-epidemic health system recovery: A comparative case study analysis of routine immunization programs in the Republics of Haiti and Liberia
Source: PLoS One. 2023 Oct 17;18(10):e0292793. doi: 10.1371/journal.pone.0292793 (PMC10581452; doi:10.1371/journal.pone.0292793)
Supplement: S4 Appendix — (DOCX) [file pone.0292793.s006.docx]

**Appendix D. Key informant quotes mapped against components of the Essential Public Health Services Framework**

| **Essential Public Health Services Framework Component** | **Haiti** | **Liberia** |
| --- | --- | --- |
| Assess and monitor population health status, factors that influence health, and community needs and assets | “Up until six years ago, CDC had a sort of shadow system of surveillance in the country that was more reliable, which is often the case, but I don’t know if they continued that. Even if they do, they still have very limited resources and the last section that can do surveillance is WHO and UNICEF. I’ve not really seen a very well-equipped surveillance system from those two organizations in the country. It’s very difficult, so for measles it has to be a big outbreak before it becomes notable, as is the case with other diseases as well. If it isn’t big, it doesn’t get observed.” (KI1) | “In times past, there were surveillance systems across the country with diseases having their own structure and system – malaria, TB, etc., and EPI would do the same with all those different diseases. But with the introduction of GPEI, we introduced a more radical structure. At the county level, there was a surveillance officer and at the national level there were surveillance officers. Then we also had a local presence in communities of interest. These resource persons were focal points such as traditional healers, spiritual healers, and the rest of it.” (KI20) |
| Investigate, diagnose, and address health problems and hazards affecting the population | “If the government doesn’t invest in their own workforce and their own program, external programs can’t fund them forever. But the capacity of people who are there – it’s really good, they know what they’re doing and the information. But some of them were complaining that they don’t have a car to go investigate a case of measles, or they don’t have a computer. The minimum requirements of them doing their job are not available to them. That leads to more weakness. The coverage in Haiti just went down again as well because there’s no sustainability in the funding.” (KI9) | ““The good thing about EVD is that when it [came] to surveillance, the establishment of the surveillance system was interconnected. When EVD surveillance happened, there was support for communication and whatnot. Specimens would go to the lab to get tested and we would hear about that during our daily case counts. And through that, we could hear about polio conditions and immunization conditions. So now, that system in place helps us and…captures all reportable diseases.” (KI16) |
| Communicate effectively to inform and educate people about health, factors that influence it, and how to improve it | “If you look at the coverage level of routine vaccines immediately after…they had issues before the earthquake, but the earthquake just exacerbated them. So, they started low and then they just got lower…There was a whole measles, rubella, oral polio, Vitamin A, integrated campaign immediately after the earthquake, and then 2 years later as well…But we know that strengthening routine immunization takes longer – years. You can’t do it overnight. But campaigns are a quick success and people want a quick win. But, also, some people are hard to reach, and they can’t be reached by campaigns or routine immunization due to a rural area or distance or whatnot.” (KI9) | ““There was a UN mission in Liberia [with] a radio station that provided wide coverage, but not everyone in the rural area has a radio or the capacity to buy batteries, because there’s no electricity in most areas,” recalled KI14. “So, in rural areas, the government had to rely on local leaders, elders, women groups, tribal leaders, civil society groups. That challenge in communication in rural versus urban [areas] was addressed by having a centralized channel for disseminating messages with an incident management team in place deciding on the messages going out…There was a bottom-up and top-down approach mutually reinforcing the messaging.” (KI14) |
| Strengthen, support, and mobilize communities and partnerships to improve health | “[Referrals to primary care providers for immunization] were identified as a good practice because when you come [to the cholera treatment unit], the family sometimes also comes, and you can go check. But what happens is that those spaces are physically distant, or there’s red tape all over. I don’t think it happened a lot where people said, ‘oh, since I’m here, I’m going to go see my status and get my second dose of whatever,’ because it was quite separated from the fear of cholera contamination…So yes, referral would have been great, and it was identified as such, but it didn’t happen often.” (KI5) | “The immunization program is one of the oldest programs in the Ministry. They’ve been involved in primary health care for more than four decades now. They’ve done a lot of work around awareness creation and their messages on the issue of vaccinating children have resonated very well with the population. So, when you talk about a rural parent in Liberia talking about immunization, [it’s] because there’s frequent awareness on polio and integrated campaigns – they do two or three per year.” (KI13) |
| **Essential Public Health Services Framework Component** | **Haiti** | **Liberia** |
| Create, champion, and implement policies, plans, and laws that impact health | “When you have a national health strategy, you match resources available with current priorities, determine funding gaps with priorities, and those types of tools are useful to make sure donor resources increase where funding gaps are, and make sure that donor resources align with this plan over time. Haiti was one of the main countries not interested in this kind of mapping, and we can’t push them to do it. The government wanted to do it on their own without any help, but because the exercise isn’t there, it’s tough for people to understand where the gaps are. [Ministries of Health] in other countries do this on a regular basis.” (KI6) | “Two or three months after EVD started, there were technical guidelines issued to bring the attention back to the immunization program as much as possible and reassure health workers to give them minimum measures to provide safe immunization services. Those technical guidelines were really helpful for bringing back political attention and providing some confidence in routine immunization programs.” (KI12) |
| Utilize legal and regulatory actions designed to improve and protect the public’s health | “I hear this at least ten times a year – an implementing partner complaining they only [get] two thirds of the doses they requested. So, after three years of receiving two thirds, two thirds, two thirds, a new cohort of children is created for a campaign. If facilities are only receiving two thirds of what they request, we’ll never get over 66% coverage. And that’s the maximum. We have no idea why they are getting only two thirds. I feel that [MSPP] fears to ask this question to PAHO. I would love to ask at the table, but if I ask, I could lose my job – other people have. Those people are so powerful, they can even cut the head of ministries.” (KI3) | “USAID had also started providing financial support with the Liberian government managing it directly rather than passing it through NGOs. [MoHSW] used that as a reimbursement mechanism. It had to be spent on earmarked areas identified by both governments. The [MoHSW] and Ministry of Agriculture were the main benefactors of that money, and it included bolstering immunization and primary health care.” (KI18) |
| Assure an effective system that enables equitable access to the individual services and care needed to be healthy | “The cross-cutting theme I see people grappling with again and again is that the system isn’t really a system. It’s so many little pieces, and if even one of those pieces works – like, let’s say there’s a really good primary care level facility – even if that’s working, that doesn’t mean that the next level is working. That lack of continuity and ability to communicate to the population what to expect, and where to go, and how to navigate creates this sort of perpetuated lack of trust, and lack of paying attention at all, and why even bother?” (KI7) | “There’s a schedule of, say, every Monday I have to go to this location in my catchment community and vaccinate because people cannot come to the facility, because [their] child is not sick, or they have things to do, or they have many young children that cannot be left at home. There’s no motivation or incentive to walk for hours just to get vaccinated. So, it’s the vaccinator’s responsibility to make that trip, keep on schedule and get people vaccinated. One quarter of children in our system are vaccinated by that approach.” (KI13) |

| **Essential Public Health Services Framework Component** | **Haiti** | **Liberia** |
| --- | --- | --- |
| Build and support a diverse and skilled public health workforce | “You also have to pay health resources per diem. And you hear ‘oh, it’s for their own good and it’s a benefit they should appreciate,” but you’re talking to people who most likely haven’t been paid for a year, and people who have been offended over and over again, and they want to feed their own children, and they’ll do that with the $150 for vaccinating for five days. And if you don’t pay them, they won’t do anything because they’ve been offended before.” (KI1)  “The salary is low or nonexistent for routine immunization, while the money is made from campaigns – which come from outside funders. So, the government really doesn’t give much money from themselves for immunization.” (KI9) | “We have a vaccinator cadre of HCWs – they are like nurse aides, but they haven’t undergone formal training…No one knows how they get paid. Sometimes they sacrifice for weeks or months, but you don’t get on payroll for one, two, or three years and sometimes just on a part-time basis. Those who hire you are not involved in the payroll process. Ten percent of vaccinators are contractors – not on government payroll – and they’re hired by [a] facility to be paid by NGOs. So once the project ends, they stay and hope they get on payroll.” (KI13)  “It is a serious challenge. We are heavily donor driven. There is also some issue with transparency for funding by partners. UNICEF is providing funding to pay community health assistants while World Bank is providing funding, [so] why are these people not getting paid? When funders pull out, how are we going to find funding for them? The government has very scarce resources and we are not ready to pay community health assistants on the payroll.” (KI19) |
| Improve and innovate public health functions through ongoing evaluation, research, and continuous quality improvement | “There are quality issues; not everyone is collecting data the same way. We aren’t collecting it for a census, we’re just collecting it to help a program. Microplanning comes from the base of pyramid, and the base struggles.” (KI5)  “The last census we had in Haiti was in 2003 or 2005. That was about 20 years ago. The only thing we were able to do as a country was apply formulas to estimate present and future population. We don’t have the real population of Haiti, though – we’re just applying a percentage to estimate the births. We need to actually count the amount of children every year or so to estimate vaccination coverage.” (KI3) | ““We do not have a very effective population registry system.…The population is changing, but we use a growth rate that varies from time to time and is not the same across counties or communities because of population migration and other factors. It’s a big challenge to determine actual coverage since we’re using a denominator from 2008. Once we have a good birth registration system or a good health information system that is universal or tracking 98% or 95% of deliveries, then we can say, look, our estimate should be based on deaths under one and deliveries as the basis for our denominator. We have ten to fifteen percent of women delivering outside health facilities, though…If you do not know how many children are born or die, then you cannot make a good estimate. So, coverage is likely different than what actually comes out of national surveys.” (KI13)  “There was a lot of push data that was generally accurate. But the books didn’t let you easily figure out who was missing what…So, vaccinators had to go line by line to say, this kid is nine months old, circle his name, and now I have to go find him.” (KI11) |
| **Essential Public Health Services Framework Component** | **Haiti** | **Liberia** |
| Build and maintain a strong organizational infrastructure for public health | “Haiti has been in crisis mode for so long that I’m not sure if the cholera outbreak changed that much. Things were barely functioning, and people were distracted before, anyways. If anything, cholera brought attention and resources into a regularly bogged down system. It was an incredible opportunity that wasn’t captured super well…The biggest opportunity missed was that there were so many organizations working on stuff, and everyone had an agenda just for their thing, and resources just for their thing. And if those resources had been pooled, there could have been a much more concerted effort to invest in infrastructure, including facilities, and strategy for workforce, and all of that, but money wasn’t shared or organized.” (KI7) | “We had people in health communications, logistics, [and] supply chain all falling under the incident management system that [was] a replica of the CDC system,” shared KI21. “We had Liberian leadership within each thematic area. EPI, surveillance, contact tracing, laboratory, health communications, case management, etcetera – those were all thematic areas. [And] we had international partners embedded in each of these thematic areas.” (KI21) |
