# Supplementary material for: Post-epidemic health system recovery: A comparative case study analysis of routine immunization programs in the Republics of Haiti and Liberia
Source: PLoS One. 2023 Oct 17;18(10):e0292793. doi: 10.1371/journal.pone.0292793 (PMC10581452; doi:10.1371/journal.pone.0292793)
Supplement: S5 Appendix — (DOCX) [file pone.0292793.s007.docx]

**Appendix E: Historical Contexts of Haiti and Liberia**

These historical summaries were developed largely independently of the data collection and analytical approaches described in the methods section, which focused explicitly on gathering data pertaining to immunization program recovery. Rather, these summaries are meant to provide relevant contextual details and inform broader understandings of health system functioning in Haiti and Liberia.

***Haiti***

The recent trajectory of Haiti’s routine immunization efforts is a consequence of the country’s tumultuous history as much as it is a consequence of the 2010 earthquake and 2010s cholera epidemic. In 1791, an insurrection led by Toussaint Louverture and other enslaved Haitians sparked a long and bloody revolution against France – the largest slave-led revolt since Spartacus’ rebellion against the Roman Empire nearly 2,000 years prior [1]. Haiti emerged victorious in 1804 as the first Caribbean nation to liberate itself from its colonizer, but the aftermath of the revolution plunged the country back into centuries-long turmoil. The French monarch, Charles X, deployed armed forces to Haiti, forcibly demanding 150 million francs from the new nation to indemnify its former colonizers against lost revenues from slavery. This act of extortion mired Haiti in debt, decimated its economy, and erased its political autonomy in all but name. Haiti finally serviced its full debt to France in 1947, by which time it had paid back more than twice the original sum owed: the modern equivalent of over USD$20 billion [2,3].

The United States’ interference in Haitian affairs further set the stage for the country’s present-day health system woes. In the late 1800s, the U.S. grew increasingly interested in annexing Haiti to “secure a defensive and economic stake in the West Indies” – a strategy that came to fruition in 1915, when a mob murdered Haiti’s then-President Vibrun Guillaume Sam in Port-au-Prince [4]. The U.S. – another of Haiti’s major creditors, in addition to France – quickly seized control of the National Bank of Haiti and unilaterally transferred millions of dollars’ worth of gold to the National City Bank of New York, claiming it as partial repayment of Haiti’s debts [5]. President Woodrow Wilson then ordered the U.S. Marines to occupy Port-au-Prince on grounds of protecting U.S. foreign interests [4]. The Haitian-American Convention,^[[1]](#footnote-1)^ ratified in September 1915, subsequently granted the U.S. authority to control Haiti’s public finances for the next ten years and appoint a sanitation engineer to oversee public health and development activities [6]. “To Belgium's Congo, to Germany's Belgium, to England's India and Egypt, the United States has added a perfect miniature in Haiti,” wrote the American journalist Herbert J. Seligmann in 1920 [7].

The American occupation of Haiti – a violent, militarized enterprise masquerading as a benevolent humanitarian mission – lasted until 1934, during which time the U.S. Navy and Marines ineptly attempted to modernize Haiti’s public health and sanitation infrastructure. The military’s initial efforts to study tropical diseases, construct sanitation systems, build hospitals, and provide medical care garnered minimal institutional and financial support from the U.S. government, and did little to improve living conditions for the vast majority of Haitians.[8] In light of these failures, the U.S. solicited the Rockefeller Foundation to conduct a major health survey of Haiti. The survey met with considerable resistance from the Haitian people and established precedents for enlisting nonstate actors as instruments of state interests and claiming the mantle of development as pretext for continued occupation.[9] The so-called process of “Haitianization” eventually began in 1931, whereby the U.S. gradually relinquished control of the country’s public institutions to the Haitian government – but in doing so, sabotaged the country’s fledgling health system. By order of the U.S.-appointed Sanitation Engineer, health budgets were slashed, leaving patients to pay for medicines and health supplies out-of-pocket [8].

Over time, the deliberate, methodical efforts of state and nonstate actors to atrophy Haiti’s public sector capacities have led the country to be dubbed “a republic of NGOs” [10]. In 2011, for example, as much as 99% of all aid earmarked for Haiti went to the estimated 10,000 active humanitarian agencies, contractors, and other nonstate service providers operating in-country, with a mere 1% channeled toward the national government or other public institutions [11]. Connecting the legacy of Haiti’s colonization to the current state of its routine immunization efforts, KI1 shared:

“Haiti is the first independent country [in the Caribbean] and they’re very proud of that. You have to respect that and navigate the hate-love relationship with the international community. That’s the cultural. The political is linked to that. It’s the whole dynamic of the U.S. on one side and [the Pan-American Health Organization, PAHO] on the other side, and the different NGOs that are present…It’s not the core business of public health people that want to maintain a 95% immunization rate. That’s not the political agenda behind routine immunization or a campaign, [but] you end up in a political arena that is dealing with issues that have nothing to do with public health. That is, as I’ve seen, the core issue with immunization.”

The Pan-American Health Organization (PAHO) has affirmed these observations about Haiti’s singular historical trajectory, writing, “Although it is making a consistent effort to join regional institutions, few if any other members share the same challenges or background, or even understand its culture. Haiti is not fully integrated as an equal member in the Caribbean or Latin America. It is an orphan without siblings, but with many foster parents” [12].

Following a 2004 coup wherein former President Jean-Bertrand Aristide was ousted from power, the United Nations issued a resolution to establish the Mission des Nations Unies pour la stabilisation en Haïti (MINUSTAH) [13,14]. MINUSTAH was a peacekeeping mission comprised of militarized forces mandated to restore the rule of law and strengthen public institutions – a measure that echoes Haiti’s long history of subjugation by external powers and annexation of its state functions. In 2009, the year before its earthquake, Haiti was ranked among the most corrupt countries in the world [15]. Its weakened public sector meant that critical services (i.e., sanitation, healthcare, education, military, and fire safety) were either nonexistent or outsourced to private and civil society actors, and only a small police force comprised its disaster response workforce [16].

***Liberia***

Like Haiti, Liberia’s colonial roots serve as prologue to its present-day health system challenges. Originating as a project of the American Colonization Society (ACS) – a U.S. group that advocated for the repatriation of free Black Americans to Africa – Liberia first consisted of settlements affiliated with individual American states, including Mississippi-in-Africa, New Georgia, Kentucky-in-Africa, and Maryland-in-Africa, among others [17]. Support for the Society’s mission stemmed, in part, from a desire to minimize the U.S. population of free Black Americans and avert potential slave rebellions. In a striking parallel to America’s occupation of Haiti nearly a century later, many ACS leaders also envisioned themselves as humanitarians, albeit acting under divine mandate. Nevertheless, they continued dispatching freedpeople to Africa despite full awareness that Americo-Liberians were dying in large numbers from tropical infectious diseases [18,19].

In the 1940s, the U.S. launched a Public Health Mission in Liberia to tackle the country’s high prevalence of infectious diseases, reorganize the country’s then-Bureau of Public Health and Sanitation, and train the local health workforce [20]. The purpose of these missions – which were civilian in name but militarized in their execution – was to “survey Liberian natural resources and create a healthy environment for postwar development and export through an American-controlled harbor” [21]. Asserting that a fully autonomous Liberia would collapse into financial ruin after World War II and contribute little to the global postwar economy, the U.S. later sought to manipulate Liberian domestic reforms by controlling the flow of aid into the country, thereby "developing capacities of the people for whose benefit it was intended” [21].

Two modern-era health crises have played a direct role in shaping Liberia’s post-Ebola routine immunization successes: the First and Second Liberian Civil Wars. During the First Civil War (1989-1997), Samuel Doe – the Liberian president and former general who had seized power through a violent coup – was brutally executed by a rebel leader, Prince Johnson. Following a power struggle between Johnson’s forces and another faction led by Charles Taylor, a ceasefire in 1995, and a national election in 1997, Taylor was eventually inaugurated as the 22^nd^ President of Liberia [22]. Just two short years later, however, the Second Civil War erupted. Two rebel militia groups – Liberians United for Reconciliation and Democracy and the Movement for Democracy in Liberia – emerged in the northern and southern regions of the country, respectively, before usurping power from Taylor [23]. After years of violence, Taylor’s resignation, and months of negotiation, the warring factions signed the Accra Comprehensive Peace Agreement in 2003, ushering in a two-year transitional regime led by Gyude Bryant [24]. Women of Liberia Mass Action for Peace – a movement organized by Leymah Gbowee, Crystal Roh Gawding, and Comfort Freeman – was a major driving force behind brokering the 2003 peace agreement [25]. This movement was also instrumental in electing Ellen Johnson Sirleaf, Africa’s first female head of state, to the Liberian presidency in 2005.

Years of civil war wrought devastation upon Liberia’s public health and healthcare infrastructure. Of the country’s 550 pre-war health facilities, only 233 functional public facilities (12 hospitals, 32 health centers, and 189 clinics) and 121 functional private facilities (10 health centers and 111 clinics) survived the conflict [26]. The wars had also decimated Liberia’s health workforce: in 2002, there were only 4,000 full-time and 1,000 part-time staff serving a population of roughly 3 million people, including 168 physicians, 273 physician assistants, 453 registered nurses and more than 1,000 nurse aides and other health professionals – far short of the WHO-recommended minimum health workforce density of 4.45 doctors, nurses, and midwives per 1,000 people [27,28].

**References**

1. D’Amato, P. Who Was Spartacus? Available online: http://socialistworker.org/2010/01/15/who-was-spartacus (accessed on 20 February 2022).

2. Daut, M. When France Extorted Haiti – the Greatest Heist in History Available online: http://theconversation.com/when-france-extorted-haiti-the-greatest-heist-in-history-137949 (accessed on 20 February 2022).

3. Ata, T. Haiti’s Debt to France. *ILLUMINATION* 2020.

4. U.S. Department of State U.S. Invasion and Occupation of Haiti, 1915-34 Available online: https://2001-2009.state.gov/r/pa/ho/time/wwi/88275.htm (accessed on 20 February 2022).

5. Bauduy, J. The 1915 U.S. Invasion of Haiti: Examining a Treaty of Occupation. *Soc. Educ.* *79*, 244–249.

6. Pezzullo, R. *Plunging Into Haiti: Clinton, Aristide, and the Defeat of Diplomacy*; 1st ed.; University Press of Mississippi: Jackson, MS, USA, 2006;

7. Seligmann, H.J. The Conquest of Haiti. *The Nation* 1920.

8. Lopez, P.J. Clumsy Beginnings: From ‘Modernizing Mission’ to Humanitarianism in the US Occupation of Haiti (1915–34). *Environ. Plan. Econ. Space* **2015**, *47*, 2240–2256, doi:10.1177/0308518X15598262.

9. Reichardt, E.M. *The Role of the Rockefeller Foundation in the Origins of Treponematosis Control in Haiti, 1915-1927*; Rockefeller Archive Center Research Reports; Rockefeller Archive Center: Sleepy Hollow, NY, USA, 2021; p. 19;.

10. Office of the Special Envoy to Haiti *Has Aid Changed? Channelling Assistance to Haiti before and after the Earthquake*; United Nations Office of the Special Envoy for Haiti: New York, NY, USA, 2011;

11. Kristoff, M.; Panarelli, L. *Haiti: A Republic of NGOs?*; United States Institute for Peace: Washington, D.C., 2010;

12. de Ville de Goyet, C.; Sarmiento, J.P.; Grünewald, F. *Health Response to the Earthquake in Haiti: Lessons to Be Learned for the next Massive Sudden-Onset Disaster*; Pan American Health Organization: Washington, D.C., 2010;

13. United Nations Peacekeeping MINUSTAH Fact Sheet Available online: https://peacekeeping.un.org/en/mission/minustah (accessed on 20 February 2022).

14. UN Security Council Resolution 1542 (2004) / Adopted by the Security Council at Its 4961st Meeting, on 30 April 2004 2004.

15. Transparency International Corruption Perceptions Index Available online: https://www.transparency.org/en/cpi/2010 (accessed on 20 February 2022).

16. Kirsch, T.; Sauer, L.; Sapir, D.G. Analysis of the International and US Response to the Haiti Earthquake: Recommendations for Change. *Disaster Med. Public Health Prep.* **2012**, *6*, 200–208, doi:10.1001/dmp.2012.48.

17. Alexander, A. *A History of Colonization on the Western Coast of Africa*; 1846;

18. McDaniel, A. Extreme Mortality in Nineteenth-Century Africa: The Case of Liberian Immigrants. *Demography* **1992**.

19. Schick, T.W. *Behold the Promised Land: A History of Afro-American Settlers in Nineteenth-Century Liberia*; The Johns Hopkins University Press: Baltimore, Maryland, USA, 1980;

20. Poindexter, H.A. The United States Public Health Mission in Liberia. *J. Natl. Med. Assoc.* **1950**, *42*, 4.

21. Beecher, L.N. Second World War and U.S. Politico-Economic Expansionism: The Case of Liberia, 1938–45 | Diplomatic History | Oxford Academic. *Dipl. Hist.* **2007**, *3*, 391–412, doi:https://doi.org/10.1111/j.1467-7709.1979.tb00324.x.

22. Miller, T.C. Firestone and the Warlord: Tough Talk in the Jungle. *ProPublica* 2014.

23. Kieh, G.K. The Roots of the Second Liberian Civil War. *Int. J. World Peace* **2009**, *26*, 7–30.

24. Bryant Takes Power in Liberia. *The Guardian* 2003.

25. Kuwonu, F. Women: Liberia’s Guardians of Peace Available online: https://www.un.org/africarenewal/magazine/april-2018-july-2018/women-liberia%E2%80%99s-guardians-peace (accessed on 20 February 2022).

26. Government of Liberia; World Health Organization Liberia: Health Situation Analysis Final Report, Jul 2002 - Nov 29, 2003 2003.

27. Academy for Educational Development Assessment of Health Training Institutions in Liberia 2007.

28. Global Health Workforce Alliance; World Health Organization A Universal Truth: No Health Without a Workforce 2014.

1. Article XIII of the Haitian American Convention states: “The Republic of Haiti, being desirous to further the development of its natural resources, agrees to undertake and execute such measures as in the opinion of the high contracting parties may be necessary for the sanitation and public improvement of the Republic under the supervision and direction of an engineer or engineers, to be appointed by the President of Haiti upon nomination by the President of the United States, and authorized for that purpose by the Government of Haiti.” [↑](#footnote-ref-1)
